# Predicting which patients with cancer will see a psychiatrist or counsellor from their initial oncology consultation document using natural language processing

**DOI:** 10.1038/s43856-024-00495-x

**Published:** 2024-04-08

**Authors:** John-Jose Nunez, Bonnie Leung, Cheryl Ho, Raymond T. Ng, Alan T. Bates

**Affiliations:** 1 BC Cancer, Vancouver, BC Canada; 2 Department of Computer Science, University of British Columbia, Vancouver, BC Canada; 3 Department of Psychiatry, University of British Columbia, Vancouver, BC Canada

**Keywords:** Medical research, Prognosis, Computational biology and bioinformatics, Cancer

## Abstract

**Background:**

Patients with cancer often have unmet psychosocial needs. Early detection of who requires referral to a counsellor or psychiatrist may improve their care. This work used natural language processing to predict which patients will see a counsellor or psychiatrist from a patient’s initial oncology consultation document. We believe this is the first use of artificial intelligence to predict psychiatric outcomes from non-psychiatric medical documents.

**Methods:**

This retrospective prognostic study used data from 47,625 patients at BC Cancer. We analyzed initial oncology consultation documents using traditional and neural language models to predict whether patients would see a counsellor or psychiatrist in the 12 months following their initial oncology consultation.

**Results:**

Here, we show our best models achieved a balanced accuracy (receiver-operating-characteristic area-under-curve) of 73.1% (0.824) for predicting seeing a psychiatrist, and 71.0% (0.784) for seeing a counsellor. Different words and phrases are important for predicting each outcome.

**Conclusion:**

These results suggest natural language processing can be used to predict psychosocial needs of patients with cancer from their initial oncology consultation document. Future research could extend this work to predict the psychosocial needs of medical patients in other settings.

## Plain language summary

Patients with cancer often need support for their mental health. Early detection of who requires referral to a counsellor or psychiatrist may improve their care. This study trained a type of artificial intelligence (AI) called natural language processing to read the consultation report an oncologist writes after they first see a patient to predict which patients will see a counsellor or psychiatrist. The AI predicted this with performance similar to other uses of AI in mental health, and used different words and phrases to predict who would see a psychiatrist compared to seeing a counsellor. We believe this is the first use of AI to predict mental health outcomes from medical documents written by clinicians outside of mental health. This study suggests this type of AI can predict the mental health needs of patients with cancer from this widely-available document.

## Abstract (web summary)

Nunez et al. investigate the use of natural language processing to predict which patients with cancer will see a psychiatrist or counselling using the initial oncology consultation document. Their study supports the use of such techniques with widely-available medical documents to better address the psychosocial needs of cancer patients.

## Introduction

Cancer is not only a leading cause of death, but a disease that substantially impacts physical, mental, and social health^1^. Patients with cancer have an increased risk of developing mental illnesses following diagnosis^2^. Approximately one-third of patients with a mental health condition before cancer diagnosis are at particular risk for worsened distress^2^. Cancer can impact employment and relationships^3–5^, adding more strain to a patient’s financial, interpersonal, and support systems. Conditions such as depression and anxiety not only degrade quality-of-life, they are associated with decreased rates of survival, possibly by impacting a patient’s ability to follow through with treatment^6–8^. To help address psychosocial needs, cancer centres employ clinicians such as psychiatrists and counsellors specializing in psychosocial care for people with cancer^9^.

Despite the development of psychosocial oncology as part of cancer care, patients with cancer continue to have unmet psychosocial needs^10–12^. Achieving equity-oriented healthcare in cancer will require better support for patients with psychosocial needs including comorbid mental illness^13^.

While lack of resources often contributes to unmet needs, there is also evidence failure to detect psychosocial needs plays a role, especially in high-resourced settings^14^. Prior work has found treating oncologists could only identify around one-third of severely distressed patients, and did not refer patients to psychosocial resources effectively^15,16^. This may be due to treating oncologists being focused on cancer control, having time constraints, using close-ended questions, and/or having cultural and socioeconomic differences from their patients. In addition, patients may not know resources are available, or be reluctant to share their difficulties^17^.

Machine learning (ML) can train models to predict outcomes such as which patients could benefit from a referral to a psychiatrist or counsellor. Such models could then be incorporated into an EMR and flag certain patients. ML models can incorporate structured data, which has been processed into specific features such as genetic markers, demographic features, or comorbidities. However, the availability of structured data can vary between cancer centres, which may limit their widespread use^18–21^. Using structured data can also limit the types of data that can be used, as not all data can be easily extracted or structured; a centre may record the marital status of their patients, but not whether they are currently having relationship difficulties^22^.

Using unstructured data, such as the initial oncology consultation document, can address some of these drawbacks. Unstructured data may possess information relevant for predicting whether a patient will see a psychiatrist or counsellor that may not be routinely stored as structured data. As most patients being treated for cancer would have an initial oncology document, a model using this data could be widely used, no matter what other data a cancer centre records.

Using ML to predict outcomes from documents falls under the branch of artificial intelligence called Natural Language Processing (NLP). Recent advances in NLP have incorporated neural networks like transformers^23^, such as those used by the recently released question-answering system ChatGPT^24^. Neural NLP models are more complex than the traditional linear methods, and are better able to understand how words in a document relate to each other, even if not directly adjacent.

Traditionally, physicians have sought to understand the psychosocial needs of patients with cancer through clinical interviews or questionnaires^25–30^. We were unable to find relevant prior work seeking to use computational methods to predict the psychosocial needs of patients with cancer. A recent study used a statistical model and structured data to forecast the number of patients with cancer and high symptom complexity a clinic would see^31^. However, this study did not make predictions for individuals.

NLP has been used in psychiatry with a variety of documents, including patient transcripts^32^ and social media posts^33,34^. Prior work using medical documents has often sought to extract data such as patient diagnoses^35–39^. Some studies have used non-neural NLP to predict readmission from discharge summaries^40,41^. Much of the recent application of NLP in mental health has used a set of 816 discharge summaries to identify the lifetime severity of a patient’s mental illness^42–50^. We did not find NLP literature predicting psychosocial outcomes from non-psychiatric medical documents, or find prior work using neural NLP to predict future psychiatric outcomes. There has been more NLP work in oncology^51–54^, including our recent work predicting survival from oncologist consultations^55^.

In this work, we investigate using NLP with initial oncology consultation documents to predict which patients with cancer will see a psychiatrist or counsellor within one year. To the best of our knowledge, predicting psychosocial needs from non-psychiatric medical documents is a novel application of NLP. Our relatively large dataset, drawn from over 50,000 patients with cancer, allows us to investigate more advanced NLP tools, including those using large language models and other neural networks, which have rarely been used in medical applications. The initial oncology consultation document is readily available, and may have relevant information for predicting psychosocial needs. We hypothesized NLP models could predict these outcomes with balanced accuracy (BAC) and receiver-operating-characteristic area-under-curve (AUC) above 0.65, a threshold exceeded in predictive work using ML elsewhere in psychiatry, such as research in depression^56,57^, suicide^58^, and bipolar disorder^59^. In this study, we train and evaluate traditional and neural models to predict which patients will see a psychiatrist or counsellor based on their initial oncology consultation document. Despite these documents not focusing specifically on psychosocial health, our best models achieve BAC above 70%, and AUC above 0.75, for both tasks when evaluated on an internal holdout test set.

## Methods

The University of British Columbia BC Cancer Research Ethics Board provided approval for this prognostic study (H17-03309), and exempted this work from requiring informed consent from participants as it was not feasible to obtain. We report this study following the Transparent Reporting of a Multivariable Prediction Model for Individual Prognosis or Diagnosis (TRIPOD) guidelines^60^.

### Data source and study population

We selected our study cohort from the 59,800 patients at BC Cancer starting cancer care between April 1, 2011 and December 30, 2016. Patients were seen for malignant disease or for non-malignant or precancerous disease requiring specialist cancer care. BC Cancer provides most cancer care in British Columbia, and is affiliated with all radiation oncologists and over 85% of medical oncologists in the province. BC Cancer provides care at six geographically diverse settings, and oversees systemic therapy at the majority of the smaller Community Oncology Network locations. BC Cancer provided our data. Clinicians generated the documents by a combination of dictation and free text processing, without explicit document structure requirements. Documents generally followed typical formatting conventions for medical consultation documents, such as including sections on identifying information, history of presentation, medical and other histories, physical examination, impression/assessment, and recommendation/plan.

### Data selection and preparation

As in our recent study^54^, we excluded participants with more than one cancer diagnosis and required patients to have at least one valid medical or radiation oncologist consultation document within 180 days of diagnosis. For this work, we used the oncologist document closest to a patient’s diagnosis.

We preprocessed documents before they were used by our models, as outlined in Note SN1. This included text tokenization for our Bag-of-Words (BoW) models, where words have their endings removed. We generated labels based on patients having a document generated by psychiatry or counselling after seeing the patient, within the 12 months following creation of their initial oncology consultation document.

### Natural language models

**Fig. 1:**
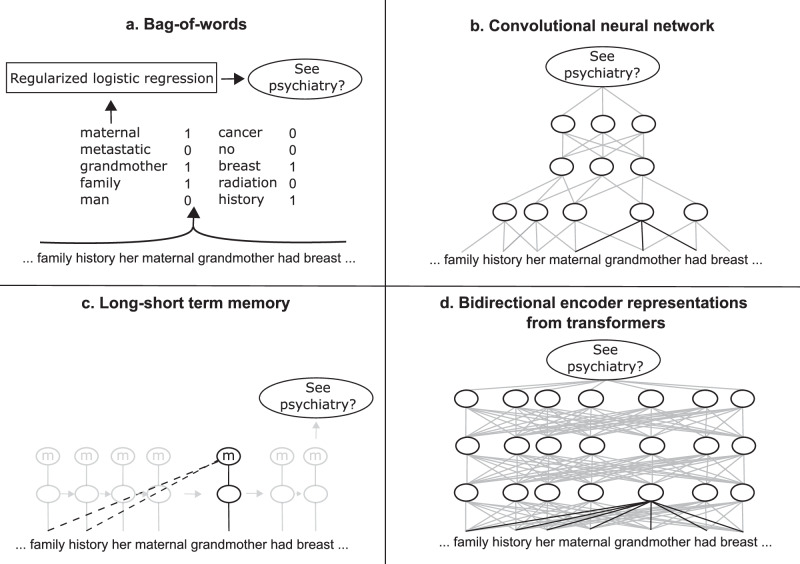
Simplified diagrams of the language models used in this work. **a** The bag-of-words model counts word occurrences in a document, which is then used by a traditional machine learning algorithm. **b** The convolutional neural network model understands a document in small adjacent clusters of words called convolutions (one is shown with black lines). The model can then learn to predict from combinations of these convolutions. **c** The long short-term memory model updates the prediction by reading the document one word at a time. It has a memory cell that allows it to remember some prior context (dotted lines). In this work, we used a bidirectional implementation, which combines the forward long short-term memory layer shown with another layer reading words in reverse order. **d** The bidirectional encoder representations from transformers model can understand how each word is connected to all other words in the document but can only read small portions of text. One word’s possible connections are indicated by a black line.

NLP models understand language based on the probabilities of which words follow each other^61^. We compared four language models: the traditional non-neural method BoW^62,63^, and three models using neural networks: convolutional neural networks (CNN)^46,47,64^, long-short term memory (LSTM)^65^, and a more recent large language model, Bidirectional Encoder Representations from Transformers (BERT)^66–68^. Figure 1 shows simplified diagrams of some of the differences in how these models understand text. Full diagrams of the model architectures can be found in their original work and elsewhere^46,61,62,64–66^. We describe further details including libraries used, class-imbalance handling, and code availability in Note SN1. To investigate whether the models were performing trivial predictions, we compared the performance of these models with a rule-based method that predicts a patient will see a psychiatrist if the consult contains the token “psychiatrist” and will see a counsellor if it contains the token “counsel”. For this rule-based method, we used the same data processing and vectorizer as for BoW. To investigate the impact of BERT having a limited number of tokens it can intake, we also investigated a variation of BERT called Longformer^69^, evaluating this model alongside CNN and BERT with different numbers of tokens. Longformer can use documents up to 4096 tokens in length, more than BERT’s limit of 512, due to having a less densely-connected self-attention. We trained Longformer using undersampling due to technical constraints, and compared it to BERT and CNN also trained with this method.

### Statistical analysis

The primary outcome was model performance when predicting whether patients would see a psychiatrist or counsellor within 12 months. We sought to avoid overfitting, when a model performs well on training data but not new data^70^, and so first randomly separated our data into training (70%), development (10%) and testing (20%) sets, a standard practice in NLP^71^. We then tuned and developed our models using only the training and development sets. For the neural models, training a model requires multiple passes through the training data to optimally train, called *epochs*. As the models will eventually overfit the training data, we continued this training until there were no further improvements in balanced accuracy for five epochs (patience) when the models were evaluated on the development set. We then compared hyperparameters based on these best performances to choose the best set of hyperparameters. To generate the final results, we continued to use standard practice, and used these tuned hyperparameters as above, stopping training of the neural models based on development set performance, and evaluating these best models on the holdout testset. To be able to provide an estimate of performance variance, we repeated this process for a total of ten times per model and target, keeping the hyperparameters unchanged, but shuffling the training data. To compare mean model performances, we conducted two-tailed dependent *t*-tests, with Bonferroni correction for multiple-comparison at 95% confidence, and calculated effect size using Cohen’s d. We used one-tailed *t*-tests to compare the rule-based method’s results with the model performances. We conducted the *t*-tests with sample size of 10, as described above; their results could be impacted by increasing the sample size, which could be done without limit. We also used a simple regression model to investigate the impact of maximum tokens on Longformer performance. We describe metrics in Table ST1.

### Interpreting our models

We measured what words were important for our BoW models based on the models’ coefficient weights, which result from training on all documents in the training set. We used the Captum Interpretability Library for Pytorch^72^ implementation of integrated gradients (IG)^73^ for an initial understanding of our neural models. This attribution method visualizes which words in a document influence a model’s prediction. The resulting visualization is easy to understand, but can only show us how the model works one document at a time. For the interpretation shown, to preserve privacy, we use a synthesized demonstration document crafted to have similar word importance to a document from a patient not included in our dataset, generated by a gynecologic oncologist.

As the above method can only interpret a model one document at a time, we developed a new method to understand a neural model over many documents using both IG and the new topic modelling technique, BERTopic^74^. BERTopic has been recently applied to medical tasks^75,76^, and is well described elsewhere. In brief, it allows *topic modelling*, which summarizes the main topics in a large collection of documents. BERTopic does so using modern transformer-based large-language models (LLM) to form embeddings of the documents separate from the topic representations, and allows customization of its modular steps. We used the “Best Practice” values, as of August 17, 2023, for these steps. This included the default sentence transformer, *UMAP*^77^, *HDBScan*^78^, scikit-learn *CountVectorizer*^79^ and the default class-based TF-IDF^61^. We created topic representations using KeyBERT^80^ and OpenAI’s ChatGPT 3.5 Turbo model^81^, alongside the default MMR representation^82^. We provide further details on our implementation in Note SN1.

We used this new model interpretation method with one CNN model for predicting seeing a psychiatrist, and one model for predicting seeing a counsellor, using the same models as for the standard IG interpretation described above. Documents longer than 1500 tokens were trimmed off the end to this amount due to technical constraints. For this new technique, we first extracted sentences from all documents in our test set that had an average IG attribution value above 0.01, setting this value empirically based on what sentences it would extract from the gynecologic oncology document used above, again using this document as it was not in any of our datasets. We then fed this collection of documents to BERTopic, using the *nr_topics* parameter to find 20 topics to represent these sentences. We again set this parameter empirically, as a trade-off between topic specificity and interpretability. For example, if this parameter was set lower, distinct topics would start to merge into one topic, such as family cancer history and personal cancer history becoming a general history topic. To focus these results, we used sentences with positive attribution values for this analysis, given that the models default to predicting patients will not see the clinicians, and we are more interested in positive predictors. This technique could also be applied to observe topic modelling for sentences with mean low attribution values, e.g., less than 0.01.

### Reporting summary

Further information on research design is available in the Nature Portfolio Reporting Summary linked to this article.

## Results

### Patient and document selection

Our patient selection was the same as in prior work^55^. Of the 59,800 BC Cancer patients, we excluded 2784 due to starting cancer care multiple times, and 9391 due to not having a medical or radiation oncology consultation within 180 days of their cancer diagnosis. This left 47,625 patients, of which 25,428 were women (53.4%) and 22,197 were men (46.6%), with a mean age (SD) of 64.9 (13.7) years (Table 1). For our prediction targets, 662 (1.4%) of patients saw a psychiatrist, while 10,034 (21.1%) saw a counsellor, within 12 months of the initial document being generated.

**Table 1:** Characteristics of the patients in the final dataset^a^

Characteristics	Dataset (*n* = 47625)
Female	25428 (53.4)
Stage I	6505 (13.7)
Stage II	8817 (18.5)
Stage III	6227 (13.1)
Stage IV	6287 (13.2)
Unknown Stage	19789 (41.6)
Age at Diagnosis, mean (SD), y	64.9 (13.7)
Survived 12 months since document^b^	37802 (79.4)
Seen by Psychiatry in 12 months since document^b^	662 (1.4)
Seen by Counselling in 12 months since document^b^	10034 (21.1)
Months after document until seen by Psychiatry, mean (SD)^b,c^	5.3 (3.4)
Months after document until seen by Counselling, mean (SD)^b,c^	2.5 (3.1)

*SD* standard deviation.

^a^Unless otherwise indicated, data are expressed as No. (%) of patients.

^b^Since the initial oncology consultation document used in this study was generated.

^c^Of patients who saw either discipline within 12 months of document generation.

We show some characteristics of the documents used for our predictions in Table 2. The documents are evenly split between medical oncology (51.5%) and radiation oncology (48.5%). 271 clinicians generated the radiation oncology documents, while 459 clinicians generated those from medical oncology. After preprocessing, the documents had a mean number of tokens between 972 and1022, depending on the model. 95.2% of documents had more tokens than the 512 limit of BERT.

**Table 2:** Characteristics of the documents used in the final dataset

Characteristics	Documents (*n* = 47625)
Generated by Radiation Oncology, *n* (%)	24511 (51.5)
Generated by Medical Oncology, *n* (%)	23114 (48.5)
Radiation Oncology Authors (Supervising Physicians^a^)	271 (100)
Medical Oncology Authors (Supervising Physicians^a^)	459 (134)
Tokens^a^ per document for BoW, mean (SD)	973 (353.8)
Tokens^a^ per document for CNN and LSTM, mean (SD)	999 (362.6)
Tokens^a^ per document for BERT and Longformer, mean (SD)	1022 (370.6)

*BoW* bag-of-words, *SD* standard deviation, *CNN* convolutional neural networks, *LSTM* long-short term memory, *BERT* bidirectional encoder representations from transformers.

^a^As BC Cancer is a teaching centre, documents were authored by medical students, resident physicians, fellow physicians, clinical associates, and supervising physicians.

^b^Tokens are words that have been processed, which can involve splitting compound words, and removing beginnings and endings. It varies depending on the model being used.

### Predicting seeing a psychiatrist

Table 3 shows the performance of our different NLP models when predicting whether a patient will see a psychiatrist in the 12 months following their initial oncologist consultation. We evaluated the models on a holdout testset. The CNN and LSTM models achieved significantly better performance than both BoW and BERT, with BAC above 70%, AUC near or above 0.80, and large effect sizes (Tables ST2 and ST3). All models significantly outperformed the rule-based method (*p* < 0.002, Cohen’s ds > 1), which predicts based on the token “psychiatrist”, achieving balanced accuracies 6.8–18.9% higher, and AUC 0.165–0.282 higher.

**Table 3:** Model performance for predicting whether a patient will see a psychiatrist in the 12 months after patient’s initial oncology consultation document was generated^a^

Model	Accuracy	BAC	AUC	F1	Sensitivity	Specificity
Rule^b^	0.981 (0.000)	0.542 (0.000)	0.542 (0.000)	0.127 (0.000)	0.088 (0.000)	0.995 (0.000)
BoW	0.877 (0.000)	0.685 (0.000)	0.784 (0.000)	0.109 (0.000)	0.486 (0.000)	0.883 (0.000)
CNN	0.851 (0.027)	0.731 (0.017)	0.824 (0.017)	0.114 (0.013)	0.607 (0.049)	0.855 (0.028)
LSTM	0.782 (0.044)	0.724 (0.012)	0.799 (0.009)	0.088 (0.012)	0.664 (0.057)	0.784 (0.046)
BERT	0.900 (0.041)	0.610 (0.046)	0.707 (0.028)	0.087 (0.009)	0.310 (0.135)	0.909 (0.044)

*BAC* balanced accuracy, *AUC* receiver-operating-characteristic area-under-curve, *BOW* bag-of-words, *CNN* convolutional neural networks, *LSTM* long-short term memory, *BERT* bidirectional encoder representations from transformers.

^a^Data expressed as the mean (standard deviation) of these metrics over 10 identical runs training and evaluating the models.

^b^Rule-based method that predicts a patient will see a psychiatrist if the document contains the token “psychiatrist”.

### Predicting seeing a counsellor

In Table 4, we show the performance of our different NLP models when they predict if a patient will see a counsellor. This prediction is again for the 12 months following their initial oncologist consultation, using a holdout testset. CNN and LSTM models are again significantly better than BoW and BERT, and have large effect sizes (Tables ST4 and ST5). The performance is significantly lower when predicting seeing a counsellor versus seeing a psychiatrist for all models except BERT (Table ST6). All models again significantly outperformed the rule-based method (*p* < 0.003, Cohen’s ds > 1), which predicts based on the token “counsel”, achieving balanced accuracies 6.8–15.7% higher, and AUC 0.126–0.231 higher.

**Table 4:** Model performance for predicting whether a patient will see a counsellor in the 12 months after patient’s initial oncology consultation document was generated^a^

Model	Accuracy	BAC	AUC	F1	Sensitivity	Specificity
Rule^b^	0.784 (0.000)	0.553 (0.000)	0.553 (0.000)	0.230 (0.000)	0.151 (0.000)	0.955 (0.000)
BoW	0.705 (0.000)	0.697 (0.000)	0.764 (0.000)	0.496 (0.000)	0.682 (0.000)	0.712 (0.000)
CNN	0.732 (0.027)	0.710 (0.005)	0.784 (0.001)	0.516 (0.005)	0.674 (0.058)	0.747 (0.050)
LSTM	0.716 (0.038)	0.706 (0.005)	0.780 (0.003)	0.508 (0.010)	0.688 (0.062)	0.724 (0.064)
BERT	0.683 (0.043)	0.621 (0.050)	0.679 (0.066)	0.394 (0.088)	0.513 (0.186)	0.728 (0.098)

*BAC* balanced accuracy, *AUC* receiver-operating-characteristic area-under-curve, *BOW* bag-of-words, *CNN* convolutional neural networks, *LSTM* long-short term memory, *BERT* bidirectional encoder representations from transformers.

^a^Data expressed as the mean (standard deviation) of these metrics over 10 identical runs training and evaluating the models.

^b^Rule-based method that predicts a patient will see a counsellor if the document contains the token “counsel”.

### Impact of token limits on transformer models

In Table 5, ST7 and ST8 we show a comparison of BERT and Longformer performance when predicting which patients will see a psychiatrist, with different maximum numbers of tokens used by a model for each document. We also show the performance of CNN for comparison. All models in this table used undersampling for class-imbalance due to technical constraints and for consistency. We see a numerical trend that more tokens leads to increases in both BAC and AUC, at least to 2048 tokens. These differences are not statistically significant on head-to-head comparison, while effect sizes were above one when comparing Longformer with 512 tokens to Longformer using 2048 or 4096 tokens. Fitting a simple regression model with number of tokens as the independent variable leads to *p*-value of 0.241, R^2^ of 0.011 for balanced accuracy, and a *p*-value of 0.062, R^2^ of 0.089 for AUC. The CNN model still has a numerically and statistically superior performance even compared to the Longformer models, except comparing the BAC between CNN and Longformer with 2048 and 4096 tokens after multiple-comparison correction.

**Table 5:** Performance^a^ of CNN, BERT and Longformer models when predicting seeing a psychiatrist when using different numbers of tokens and undersampling^b^

Model	Max Tokens	Batch Size	Accuracy	BAC	AUC	F1	Sensitivity	Specificity
Longformer	512	8	0.768 (0.152)	0.630 (0.026)	0.710 (0.020)	0.071 (0.020)	0.487 (0.180)	0.773 (0.157)
Longformer	1024	4	0.787 (0.110)	0.645 (0.040)	0.723 (0.037)	0.074 (0.018)	0.499 (0.169)	0.792 (0.115)
Longformer	2048	2	0.783 (0.044)	0.666 (0.027)	0.734 (0.018)	0.073 (0.009)	0.545 (0.085)	0.787 (0.045)
Longformer	4096	1	0.725 (0.160)	0.650 (0.035)	0.734 (0.024)	0.068 (0.018)	0.573 (0.180)	0.727 (0.165)
CNN	-	1	0.871 (0.019)	0.728 (0.012)	0.817 (0.005)	0.123 (0.012)	0.580 (0.039)	0.875 (0.020)
CNN	-	16	0.912 (0.013)	0.698 (0.020)	0.806 (0.008)	0.145 (0.008)	0.477 (0.053)	0.919 (0.014)
BERT	512	1	0.716 (0.138)	0.604 (0.034)	0.667 (0.030)	0.053 (0.009)	0.488 (0.192)	0.719 (0.143)
BERT	512	8	0.796 (0.051)	0.616 (0.020)	0.673 (0.021)	0.062 (0.006)	0.429 (0.086)	0.802 (0.053)

*BAC* balanced accuracy, *AUC* receiver-operating-characteristic area-under-curve, *CNN* convolutional neural networks, *BERT* bidirectional encoder representations from transformers.

^a^Data expressed as the mean (standard deviation) of these metrics over 10 identical runs training and evaluating the models.

^b^For results in this table, undersampling was used to deal with the class-imbalance, instead of loss weighting, due to technical constraints to run the Longformer models, and to compare with the others consistently.

### Interpreting our models

We find similarities and differences in the top ten most important tokens for our models predicting seeing a psychiatrist versus seeing a counsellor (Table 6). All tokens were used by both models, but differed in importance depending on the predictive target. We found tokens related to mental health were important in both models, including “depress” (depression, depressed) and “anxieti” (anxieties), though “anxieti” was only in the top ten for seeing a counsellor. Tokens directly related to a patient’s cancer are among the top ten most important token when predicting seeing a psychiatrist, but not a counsellor (“myeloma”, “radiat”, likely “1”). Demographic factors also seem important, such as “retir” (retiree, retired) in both, or “princ” and “georg”, corresponding to Prince George, the BC Cancer site located in northern BC, which serves a more rural population.

**Table 6:** Top ten tokens used by BoW models for predicting seeing a psychiatrist or counsellor within 12 months

Feature Importance Rank	Seeing a Psychiatrist		Seeing a Counsellor	
	Token	Coefficient Direction	Token	Coefficient Direction
1	depress	positive	counsel	positive
2	anxieti	positive	depress	positive
3	counsel	positive	anxieti	positive
4	psychiatrist	positive	princ^b^	positive
5	1^a^	positive	retir^c^	negative
6	anxious	positive	anxious	positive
7	radiat	negative	financi	positive
8	stress	positive	suicid	positive
9	matern	positive	petrov^d^	positive
10	myeloma	positive	georg^e^	positive

Feature importance was calculated from the absolute value of coefficient weights in these L2-regularized logistic regression models. Tokens are words that have had their word endings removed for processing.

*BoW* bag-of-words.

^a^The token 1 refers to the numeral, not adjacent to other letters or numbers, such as in “grade 1” or “1 pack per day”.

^b^The token “princ” refers to Prince, a common part of rural place names in British Columbia.

^c^The token “retir” referees to retired, or retiree.

^d^This token was a last name, which we have anonymized here.

^e^The token “georg” refers to George, likely referring to the northern city of Prince George.

In Notes SN2 and SN2, we show the importance of words in one synthesized document which we crafted to demonstrate similar word importance to a real patient’s document for our CNN models. This patient saw both psychiatry and counselling, which the models correctly predict. For the model predicting seeing a counsellor, a recent history of pain, and a family history of cancer in both maternal and paternal grandparents were predictive. For the model predicting seeing a psychiatrist, the maternal grandmother’s history is again important, but pain is not. Instead, we see that the oncologist writing “also noticed”, followed by additional medical symptoms, is predictive of seeing a psychiatrist.

In Tables 7 and 8, we show the results of our newly-developed technique to understand a neural model’s predictions over multiple documents, providing additional details including representative sentences in Tables ST9 and ST10. The topics cover a majority of the extracted sentence, 30,956/49760 (61.5%) for seeing a psychiatrist, and 40,424/57935 (69.8%) for seeing a counsellor. The remaining sentences are classified by BERTopic as outliers. For both targets, we find a range of topics, including those pertaining to symptoms, personal cancer history, family cancer history, substance use, and social history. As was found in the BoW interpretation, features of a patient’s cancer or treatment seem more relevant to predicting seeing a psychiatrist (topics 0, 1, 2, 3, 8, 15, 18) than for seeing a counsellor (topics 1, 3, 14, 16). Conversely, symptoms or medications used for symptom management seem more common for predicting seeing a counsellor (topics 1, 2, 8, 10, 13, 17, 19) than for psychiatry (topics 0, 5, 9).

**Table 7:** Topics of sentences that are predictive of a patient seeing a psychiatrist

Topic	Count	OpenAI Representation^a^	10-word Default BERTopic Representation
0	6411	Medical History and Mental Health	history, pain, depression, past, discomfort, anxiety, past medical, medical, medical history, abdominal
1	3606	Positive Breast Lymph Nodes	lymph, breast, node, lymph node, positive, right, mammogram, left, right breast, nodes
2	2882	Concurrent Chemotherapy and Radiation	chemotherapy, concurrent, radiation, radiotherapy, oncology, treatment, concurrent chemotherapy, medical, medical oncology, start
3	2496	Lung carcinoma mass detection	cm, mass, tumor, showed, lobe, ct, scan, right, left, fdg
4	2422	Breast Cancer Family History	cancer, family history, family, maternal, breast cancer, history, age, breast, died, mother
5	2039	peripheral edema	patient peripheral, gas, patient patient, patient, peripheral edema, peripheral, edema,,,
6	1414	Chemotherapy side effects	effects, fatigue, nausea, include, risk, nausea vomiting, vomiting, neutropenia, effects include, febrile neutropenia
7	1327	Patient work history	social, social history, works, disability, lives, today, work, history patient, currently, accompanied
8	1322	Metastatic disease scans	scan, bone scan, bone, pet, ct, pet scan, ray, ct scan, mri, disease
9	1316	absence of peripheral edema	edema, peripheral edema, peripheral, edema peripheral, lower, calf, pitting, pitting edema, edema calf, calf tenderness
10	1160	Current Medications	medications, mg, current, current medications, medications include, include, takes, medications current, taking, daily
11	1111	Patient Family Counseling Referral	patient family, patient, family, counseling, family counseling, referral, discussion, understand, today, counselling
12	1009	Menstrual History	menarche, age, menarche age, menopause, menopause age, history menarche, gynecological, gynecological history, history, menstrual
13	669	Clear Lungs Auscultation	lungs, clear, lungs clear, auscultation, clear auscultation, auscultation lungs, bilaterally, clear lungs, bilaterally lungs, entry
14	495	Normal Cranial Nerve Examination	cranial, cranial nerve, nerve, nerves, cranial nerves, normal, examination, intact, ii, examination normal
15	375	Bone Marrow Biopsy for Myeloma	myeloma, marrow, bone marrow, multiple myeloma, marrow biopsy, bone, multiple, biopsy, diagnosis multiple, diagnosis
16	308	alcohol consumption patterns	alcohol, smoking, drinks, day, quit, cigarettes, cigarettes day, pack, month, years
17	204	Baseline blood work today	blood work, work, blood, baseline, baseline blood, work today, today, cea, obtain, markers
18	201	clear lungs bilaterally	clear bilaterally, bilaterally lungs, bilaterally, lungs, lungs clear, clear, posteriorly anteriorly, anteriorly lungs, bilaterally negative, opacity left
19	189	plus documents	plus,,,,,,,,,

Topics resulting from using BERTopic on 49,760 sentences found to be positively predictive of seeing a psychiatrist according to analysis with layered integrated gradients.

^a^Representation of a topic generated by OpenAI’s ChatGPT 3.5 Turbo.

^b^Default ten-word representation generated by BERTopic, utilizing Maximal Marginal Relevance.

**Table 8:** Topics of sentences that are predictive of a patient seeing a counsellor

Topic	Count	OpenAI Representation^a^	10-word Default BERTopic Representation^b^
0	6164	Medications and Allergies	mg, medications, 30ylenol, daily, dexamethasone, allergies, mg daily, hydromorphone, taking, pain
1	6161	chemotherapy treatment plan	chemotherapy, treatment, patient, family, patient family, today, plan, cycles, given, start
2	6096	Mild intermittent abdominal pain	pain, depression, history, right, past, abdominal, left, does, difficulty, abdominal pain
3	4840	Scans for Bone Staging	scan, ct, pet, ct scan, bone, pet scan, bone scan, staging, mri, showed
4	3620	Smoking history and quitting	pack, smoking, years, alcohol, day, quit, pack year, history, cigarettes, ago
5	2436	Maternal Breast Cancer Family History	cancer, breast, maternal, breast cancer, grandmother, old, year old, family history, paternal, family
6	2087	Multifaceted Work and Social History	works, social, social history, currently, lives, history, children, work, family, prince
7^c^	2024	n, n, d, r	,,,,,,,,,
8	1632	Weight Loss Progress	pounds, lost, weight, weight loss, loss, months, appetite, pounds weight, 10, lost approximately
9	1178	Normal Cranial Nerve Examination	cranial, cranial nerve, normal, nerve, examination, oral cavity, cavity, oral, nerves, cranial nerves
10	1160	Side Effects of Chemotherapy	effects, fatigue, nausea, include, risk, alopecia, neutropenia, nausea vomiting, vomiting, limited
11	848	Varied Occupations	works, worked, work, currently, working, disability, occupation, driver, manager, worker
12	495	Menstrual history	menstrual, period, menstrual period, age, ago, menstrual cycle, gynecological, gynecological history, menopausal, premenopausal
13	473	Poor short-term memory difficulty	memory, difficulty, able, term memory, short term, short, term, word, walk, word finding
14	345	Squamous Cell Carcinoma Diagnosed	squamous, squamous cell, carcinoma, cell, cell carcinoma, diagnosis, differentiated, tongue, invasive, right
15	199	Port Cath Insertion	port, cath, port cath, require port, insertion, require, placement, cath insertion, inserted, cath inserted
16	182	Glioblastoma Frontal Lobe	glioblastoma, glioblastoma multiforme, multiforme, grade, diagnosis, temporal, lobe, frontal, left, resection
17	174	Depression and alcohol use	depression depression, depression, trazodone, depression significant, significant alcohol, alcohol intake, intake, significant, alcohol, use depression
18	159	Clear Breath Sounds	respiratory, auscultation, air entry, entry, air, reveals, exam reveals, sounds, clear, exam
19	151	Tylenol dosage and breaks	30ylenol 30ylenol, 30ylenol, half tab, tab, 30ylenol, needed 30ylenol, break, 30ylenol needed, doses, half

Topics resulting from using BERTopic on 57,935 sentences found to be positively predictive of seeing a counsellor according to analysis with layered integrated gradients,

^a^Representation of a topic generated by OpenAI’s ChatGPT 3.5 Turbo.

^b^Default ten-word representation generated by BERTopic, utilizing Maximal Marginal Relevance.

^c^This topic represents one-character sentences that are usually clinician initials.

## Discussion

In this work, we investigated the use of NLP with patients’ initial oncology consultation documents to predict whether they will see a psychiatrist or counsellor in the year following the date of the consultation document. Our best models achieved BAC over 70% and AUC over 0.80, for predicting whether they would see a psychiatrist. Performance was worse for predicting which patients will see a counsellor, though best models still achieved BAC and AUC above 70%. Two types of neural models, CNN and LSTM, outperformed the simpler BoW models. This suggests these predictions may benefit from a more complex understanding of language made possible by neural networks, in contrast to related work using similar data and techniques to predict the survival of patients with cancer survival^55^. While we could not find similar work to which we could compare these results, these metrics are comparable to or better than other applications of ML for predicting future events in psychiatry, such as predicting whether a patient’s depression will respond to an antidepressant^56,57^, whether someone will complete or attempt suicide^58^, or if a child will later develop a bipolar disorder^59^. This supports the validity of this technique for our task, and more generally, the potential use of NLP for predicting psychiatric outcomes from non-psychiatric medical documents.

Our models’ ability to better predict whether patients would see a psychiatrist versus a counsellor is somewhat surprising. The difference may not be clinically significant, but we expected seeing a counsellor to be easier to predict. Seeing a psychiatrist is more *class-imbalanced;* the ratio between those seeing a psychiatrist and not seeing one is quite extreme. Generally, ML models will perform better on tasks with less class-imbalance^83^. Our result may be due to patients seeing counsellors at BC Cancer for a variety of reasons, including both psychological assistance and social needs such as housing or transportation. It may be difficult for our models to account for these different reasons. Our results also suggest seeing a psychiatrist is more related to the medical information within the text. The BoW model had top ten features related to a patient’s cancer, while in the CNN model, normal heart rate was a negative predictor. We did not see these relationships in our model predicting which patients will see a counsellor.

Model interpretations supported they were using relevant and appropriate data to make their predictions. Important words for our BoW models included words related to mental illness, aspects of the patient’s cancer illness, and demographic factors. Interpreting a neural model from an initial oncology document not included in our dataset showed an example of how the models make their predictions. In this document, shown here by an analogous synthesized document, the CNN model used current pain and a family history of cancer to predict seeing a counsellor. The bidirectional relationship between pain and psychological health is well established^84^, while the family history may attest to intergenerational trauma associated with cancer. Similarly, for seeing a psychiatrist, the model again uses family history. It also found that “also noticed” followed by somatic symptoms supported a referral to psychiatry. This may imply the model is learning a patient endorsing many somatic symptoms may increase their chance of seeing a psychiatrist, consistent with known relationships^85^.

We furthered this initial neural model interpretation by developing a new technique to interpret neural models over multiple documents. By using BERTopic to model the topics of sentences with high mean positive attribution from IG, we see further evidence that the models are using a variety of text, including those pertaining to a family history of cancer history, and symptoms. Given this is a new technique, these results should be interpreted cautiously. However, they do seem to also suggest possible differences between the factors predictive of seeing a counsellor versus psychiatrist as suggested by our BoW prediction, such as symptoms being used more to predict seeing a counsellor, and disease characteristics more used for predicting seeing a psychiatrist. These topics may also suggest directions to explore to further our understanding of the psychosocial needs of cancer patients. For example, two topics for seeing a psychiatrist involve peripheral edema. This could be related to corticosteroid use, which can directly lead to both peripheral edema and psychiatric symptoms^86^. It also could be related to the presence of central nervous system tumours that often need these medications, and can also lead to psychiatric symptoms. We plan to further develop and validate this technique in future work, including investigating different parameters and choices for the modular steps.

This application of NLP could be used to help oncologists identify which patients may benefit from referral to counsellors or psychiatrists. It is unclear what performance we would need for such models to be used clinically; the sensitivity versus specificity of models could be adjusted depending on the application. Given that our models are trained on the status quo, where a degree of undetected and missed opportunities for referral exists, setting the models to have a higher sensitivity, at the expense of specificity, may be reasonable. Future work could seek to train or evaluate our methodology on a dataset where experts assess patients and label whether a patient should be referred to psychiatry or counselling. However, it could be difficult to manually label the thousands of patients required to effectively train neural models.

Comparing the results of the different models may provide direction to build upon our results and further improve performance. The better performance of our CNN and LSTM models compared to BoW may suggest that the more complex understanding of language that neural models are capable of may be useful for this task, as supported by our interpretations where this seems to be taking place. However, the numerical advantage of these models over BoW is relatively modest, especially when predicting which patients will see a counsellor. The use of neural language models over traditional NLP methods comes with disadvantages including increased computational cost, more difficult interpretability, and possibly privacy concerns^61,72,83,87,88^ so neural methods generally should be used when their advantage in performance outweighs these drawbacks. It may be possible to improve the performance of our models with further exploration of hyperparameter and architectural changes.

Given the recent advancement and success of transformer-based LLMs^24^, further investigation of these models may also help improve the performance of our tasks. The poor performance of BERT, which utilizes transformed-trained LLMs, was somewhat surprising, but may be due to our documents often exceeding the maximum number of tokens that can be used with this model. While the first portion of the consultation documents often document data that seems potentially relevant to our prediction, such as identifying data and symptoms, BERT may have often not had access to information usually featured towards the end of medical consultations, including past histories, assessment, and future planning. This limitation was supported by our investigation of the Longformer model, which can use up to 4096 tokens. We saw a numerical trend of increasing performance as the model could use up to 2048 tokens, enough for most documents (Table 2). However, even when able to use this larger number of tokens, this transformer-based model still did not outperform CNN. This may be due to Longformer’s sparse attention. Future work may want to investigate LLMs that have denser attention and can still utilize longer documents, such as BigBird^89^, and may want to further investigate LLMs trained specifically on clinical data^90^.

Future work could also seek to improve our models by adding other types of data, such as the responses from psychosocial questionnaires designed for patients with cancer^29^, to our training data. Alternatively, one could train separate models with rating scale data, and compare their performance to our models. However, while use of rating scales is becoming more common, such data is certainly not as ubiquitous, and possibly not as informative, as the initial oncology consultation document.

Future work will be needed to investigate the external validity of our models by evaluating them using initial oncology consultation documents from other cancer organizations. We have some evidence our models are using geographic features specific to British Columbia. This could lead to a drop in performance when used elsewhere, as could other differences such as language use, referral patterns, and treatment availability. If this is the case, our models could be further *fine-tuned*^61^ on data from a different source, which generally requires smaller amounts of data. Alternatively, our methodology could be used to train new models based on data from other sources, an advantage of us using the common and widely available data within initial oncology consultation documents. Given the possibility of LLM to improve with very large amounts of data, the best performance may be possible by training neural models on large numbers of these documents from multiple healthcare settings. We facilitated this by using a widely available document.”

Further investigation may not only help guide improvement of the models, but may also generate new hypotheses to investigate the relationship between course of illness and the need for psychosocial supports. To this end, future work may also want to investigate the performance of our models on subsets of patients, given possible differences related to age, gender, rural vs. urban setting, cancer stage, and cancer type^91^. Similarly, future work could investigate both false positives and false negatives from our models. False positives could show examples of those who would have benefited from referral to psychosocial supports, but faced barriers. False negatives could be investigated to determine whether our models are missing potential signs of impending psychosocial needs, or if patients only developed these needs at a later date. If the latter, future work could also explore predictive models that update with subsequent clinical documents generated from oncologists, such as progress notes or re-referrals.

Even if performance was perfect and external validity established, future work will also be needed to investigate possible barriers to using such techniques in clinical practice. This could include examining logistical barriers such as difficulties around incorporating predictive systems within electronic medical record system workflows. Better understanding of patient comfort around artificial intelligence being used is also needed, especially when pertaining to a sensitive topic such as need for psychosocial supports^92^. Another area of future investigation could be applying our methodology to predict psychosocial interventions in other medical settings, such as which patients on a medical ward will be referred to consultation-liaison psychiatry based on their internal medicine admission consultation.

As described above, we will need to evaluate our models on documents from other cancer care organizations to establish external validity. However, our documents do come from many providers in six geographically-distinct centres. We also acknowledge training our models on referral patterns that likely include missed referrals, making our models themselves imperfect. Additionally, while a comparison against a rule-based method solely using the tokens “psychiatrist” and “counsel” supports that models are not solely making trivial predictions based on whether oncologists are writing that they will make a referral, some of the predictions being made may be relatively simple, such as those based on whether consults include words such as “depression” or “anxiety”. We do, however, see that the top ten BoW tokens are varied, while the CNN interpretation shows an example of how the model can correctly predict a patient seeing the disciplines without obvious language, and that predictive sentences have a variety of topics. Another limitation is that some words used by our models are specific to our province, such as city names. This helps our models learn about geography-based differences, but such data would not be generalizable in other regions. As described above, we also note that our work did not explore our models’ performance on different subsets of the population such as those based on gender or cancer type; as an initial investigation, we defer this to future work. It is possible these NLP techniques may be a stronger or weaker tool, depending on the specific population.

We believe this is a novel application of NLP, as we were unable to find similar research attempting this task, or attempting to predict psychiatric outcomes from non-psychiatric medical documents generally. We believe further development will allow these techniques to improve and extend the lives of patients with cancer by helping to identify psychosocial needs that cause distress and sometimes interfere with cancer treatment.

### Supplementary information

Supplementary Information

Reporting summary

## Data Availability

We are unable to share the initial oncology consultation documents used in this work due to their number and our inability to anonymize the confidential information within them. These data are stored securely at BC Cancer. Readers can contact the corresponding author for additional information and data.

## Code availability

The computer code will be available upon publication on a public Github repository^93^. The trained BoW models will be available upon publication in this repository. Due to the possibility of neural models storing extractable private data^87,88^, we are unable to share our trained neural models publicly, but may be able to share them with interested parties pending medical ethics and institutional approval, and will be interested in exploring federated learning approaches.
