# Supplementary material for: Predicting which patients with cancer will see a psychiatrist or counsellor from their initial oncology consultation document using natural language processing
Source: Commun Med (Lond). 2024 Apr 8;4:69. doi: 10.1038/s43856-024-00495-x (PMC11001970; doi:10.1038/s43856-024-00495-x)
Supplement: Supplementary file 1 — Supplementary Information [file 43856_2024_495_MOESM1_ESM.pdf]

Predicting Which Patients with Cancer Will See a Psychiatrist or Counsellor From Their Initial Oncology Consultation Document Using Natural Language Processing

*John-Jose Nunez, MD, MSc; Bonnie Leung, MN-NP(F); Cheryl Ho, MD; Raymond T. Ng, PhD; Alan T. Bates, MD, PhD

Author Affiliations

BC Cancer, Vancouver, Canada: J.J.N, B.L., C.H., A.T.B
Department of Computer Science, University of British Columbia, Vancouver, Canada: J.J.N., R.T.N.
Department of Psychiatry, University of British Columbia, Vancouver, Canada: J.J.N., A.T.B.

*__Corresponding Author__

Dr. John-Jose Nunez, MD, MSc., FRCPC
BC Cancer and Department of Psychiatry, University of British Columbia
2215 Wesbrook Mall
Vancouver BC. V6T 1Z3 Canada
Tel: 604-822-7512, Email: johnjose.nunez@bccancer.bc.ca

Table of Contents

Note SN1: Supplementary Methodological Note 4
Obtaining Data 4
Text Processing..4
Language Models Used 4
Investigating the Impact of Number of Tokens on Performance 6
Hardware Used 6
Implementation..6
Note SN2. Visualizing word importance of CNN models used to which patients see a counsellor or psychiatrist in the 12 months following initial oncologist consultation document generation 8
Note SN2. Visualizing word importance of CNN models used to which patients see a counsellor or psychiatrist in the 12 months following initial oncologist consultation document generation, adapter for colour blindness..9
SR1: Supplementary References 10
Supplementary Tables..12
Table ST1: Definition of evaluation metrics reported in this work 12
Table ST2. Performance of all models when predicting if patients will see a psychiatrist in twelve months, with extended metrics .13
Table ST3. Statistical comparison of models when predicting if patients will see a psychiatrist in the twelve months following their initial oncologist consultation..14
Table ST4. Performance of all models when predicting if patients will see a counsellor in twelve months, with extended metrics...15
Table ST5. Statistical comparison of models when predicting if patients will see a counselor in the twelve months following their initial oncologist consultation..16
Table ST6. Statistical comparison of each model when predicting if patients will see a psychiatrist versus seeing a counselor, in the twelve months following their initial oncologist consultation..16
Table ST7. Performance Longformer, CNN, and BERT models when predicting if patients will see a psychiatrist with different numbers of maximum tokens, with extended metrics 17

Table ST8. Statistical comparison of CNN, BERT and Longformer models when predicting seeing a psychiatrist when using different numbers of tokens and undersampling. 19

Table ST9: Additional topic representations and example sentences from using our new interpretation technique with models trained to predict seeing a psychiatrist 19

Table ST10: Additional representations and example sentences from using our new interpretation technique with models trained to predict seeing a counsellor....22

Note SN1: Supplementary Methodological Note

Our methods are based upon prior work using this dataset and these neural models[1], with new tuning, and conducting multiple evaluations to estimate variance, given the increased variance associated with the class-imbalance of the seeing-a-psychiatrist (SaP) target, though seeing-a-counsellor (SaC) was not as imbalanced.

Obtaining Data

As used in this prior study, unstructured text documents were provided as extracted Microsoft Word documents from BC Cancer electronic health records, along with some structured data including diagnosis date, age, and cancer site at diagnosis. Additional structured data, specifically death dates, were obtained from the BC Vital Statistics by BC Cancer data stewards, and linked to our dataset. We also received metadata on extracted documents, including the medical speciality that generated the document, and the document type. We used this metadata in our document selection, excluding documents that were not consultation documents such as progress notes.

Text Processing

We used the same processing as in this prior work, replacing question and exclamation marks with periods, all spaces with single spaces, and we converted characters that were not alphanumeric, parenthesis, apostrophes, or punctuation with spaces. We removed automatically added text at the beginning and end of the documents that contained information such as identifying information, dates, and the names of the dictating providers and who they sent the documents to; these string patterns are available in the project's Github repository.

Language Models Used

Bag-of-word (BoW) models have a simple understanding of documents, simply counting the frequency that certain words occur in a document[2]. These frequencies then form a vector, which can be used by traditional machine learning algorithms. We again implemented BoW with common choices: L2-regularized logistic regression with lbfgs solver, and term frequency-inverse document frequency weighting[3]. We utilized a vector length of 5000 as smaller lengths decreased performance, and higher values did some seem to improve performance, and would lead to a high ratio features and samples. To tune the hyperparameter C, corresponding to the inverse of the lambda regularization factor, we tried values between 0.1 and 5. For SaP, we used a C of 0·6, corresponding to the inverse of the lambda regularization factor. For SaC, we used a C of 1·05. Training and evaluating a final BoW model took around 10 minutes.

When used in natural language processing (NLP), convolutional neural networks (CNN)'s use convolutions of a small number of adjacent words[2]. They then understand a document based on combinations of these small groupings, allowing a reduction of the feature space, but distant word relationships are still considered. We based our CNN models on those developed for general and medical document classification[4–6]. We used the Adam optimizer for CNN. We tried hyperparameters from these works and additional, often nearby, values. Trying different word vector length, window lengths, and output channel did not seem to benefit performance, so we used these hyperparameters as in prior work. We investigation dropout values of0.5, 0.55, 0.6, 0.65, 0.7, 0.8, 0.825, 0.85, 0.875, 0.9, 0.95, weight decays of 0, 0.1, 0.01, 0.001, 0.0001, and learning rate of 0.0001, and 0.00005. We used grid-search to investigate many combinations of these values, manually also testing some combinations with values near promising combinations Our final hyperparameter set used 300-length word vectors, window lengths of 3,4 and 5 tokens, and 500 output channels for both targets. For SaP, we used a weight-decay of 0·0001, a dropout rate of 0·85 and a learning rate of 0·0001. For SaC, we used a weight-decay of 0, dropout rate of 0·85, and learning rate of 0·00005. We trained models with up to 100 epochs, with early stopping after 5 epochs (patience) of no improvement in balanced accuracy. We used a batch size of 16, the maximum supported on our hardware. We shuffled training

data by randomly shuffling indices within our custom batch sampler. Training and evaluating a CNN model took around 25 minutes. Training and evaluating a final CNN model took around 20 minutes.

Long short-term memory (LSTM) models are a type of recurrent neural network that understands a document one-word at a time, changing the prediction at every step[2]. LSTM have memory cells that allow the model to better consider what words occurred in other parts of the document. We based our LSTM on previous models developed using regularization to avoid overfitting[7]. We used for Adam optimizer with LSTM. We used a bidirectional LSTM, where there is both a forward and backward LSTM layer, which allows each input token to be understood based on both the tokens before and after. We again tried values from prior work with additional hyperparameters. Initial investigation revealed no benefit from varying embedding lengths or hidden unit dimensions, so we used values from prior work. including word embeddings with length 300, and a hidden unit dimension of 512. We investigated dropout rates including 0.1, 0.2, 0.3, word embedding dropouts of 0.1, 0.001, and weight dropouts of 0, 0.01, 0.001, and learning rates of 0.001, 0.0001, 0.0005. We again used grid search to try many of these combinations. For SaP, we used dropout rate of 0.1, word embedding dropout of 0.01, weight dropout of 0.01, and a learning rate of 0.0001. For SaC we used dropout rate of 0.2, word embedding dropout of 0.1, weight dropout of 0.01, and a learning rate of 0.0005. We again trained models with up to 100 epochs, with early-stopping after 5 epochs (patience) of no improvement in balanced accuracy. We used a batch size of 16, the maximum supported on our hardware. We again shuffled training data by randomly shuffling indices within our custom batch sampler .Training and evaluating a LSTM model took around 4 hours.

The bidirectional encoder representation from transformers (BERT) model was developed by Devlin et al[8] to allow a deep, bidirectional understanding of language. Using state-of-the-art computational resources, they developed a transformer model which allows all pieces of text in a document to be considered at once. This model uses attention to focus on useful relationships. This model can then be fine-tuned to accomplish other tasks, such as our binary survival classification. While allowing all words to be considered with respect to how they relate to each other, these models are limited in only being able to utilize 512 tokens, which represent an entire or part of a word. We used an AdamW optimizer with BERT. During initial investigation, when there were some differences in our dataset processing and target generation, we tested different English BERT models including *bert_large_uncased*, *bert_pretrain_output_all_notes_150000* and *bert_pretrain_output_disch_100000* from ClinicalBERT[9] and *biobert_pretrain_output_all_notes_150000* and *biobert_pretrain_output_disch_100000* from BioBert[10], We seemed to achieve the best performance with *bert_base*_uncased so we proceeded with this language model. We conducted hyperparamter tuning investigating weight drops of 0, 0.1, 0.01, 0.0001, 0.00001 and learning rates of 0.001, 0.005, 0.0001, 0.0005, 0.00005, 0.00001, again using grid search for most combinations. For SaP, we used a weight decay of 0, and a learning rate of 0·001, and weight decay of 0·01, and learning rate of 0·00005 for SaC. We again trained models with up to 100 epochs, with early stopping after 5 epochs (patience) of no improvement in balanced accuracy. We used a batch size of 8, the maximum supported on our hardware. We shuffled training data by using the shuffle=True flag in the Dataloader. Training and evaluated a BERT models took around 8-12 hours.

The Longformer model was developed by Beltagy et al[11] as an extension of BERT that can handle larger documents up to 4096 tokens. Instead of BERT's memory requirements which scale quadratically with token length, Longformer's scales linearly, allowing this high limit. It does this by using more selective attention mechanisms, instead of the densely attending mechanism in BERT. Longformer instead uses three more selective attention mechanisms, a *sliding window* attending to tokens a set number away from a token, a *dilated window* allowing attention far away, and limited *global attention* which allows sense attention between only a set number of tokens. We used the pretrained model *allenai-longformer-base-4096*, a learning rate of 0.0001, a weight drop of 0, and a patience of 10. We again shuffled training data by using the shuffle=True flag in the Dataloader. Due to our limited VRAM, we were only able to run a batch size of 1 when using 4096 tokens, which led to epochs taking almost 24 hours (batch size 2 for 2048 tokens, 4 for 1024 tokens, and 8 for 512 tokens) . As such, we

used undersampling for training Longformer, which results in epochs taking less than an hour, and an entire run of training and evaluation taking around 12-24 hours.

Investigating the Impact of Number of Tokens on Performance

To investigate how the number of tokens available to a model would impact performance, we investigated the performance of Longformer using a maximum of 512, 1024, 2048, and 4096 tokens. As above, due to technical constraints we needed to use Longformer with undersampling, which we implemented as there being twice as many documents from those who did not see a psychiatrist or counsellor as those who did. Exploring this using only the training and dev sets, we found that a higher patience was required, so set this to 10. Occasionally, models would not train sufficiently to ever see an improved performance on the dev set. In this situation, we set the models to exit and not evaluate on the training set. We compared the Longformer to BERT and CNN models also using undersampling. Besides patience being set to 10, we did not change the CNN hyperparameters as their performance on the development set was comparable to when using loss-weighting. BERT did not successfully train, as judged by achieving dev set performance above 0.5, when using undersampling and the hyperparameters above. Instead, we used one of the next best performing hyperparameter combinations when tuning originally, which also happened to be the hyperparameters tuned for Longformer, a learning rate of 0.0001 and a weight drop of 0.

Hardware Used

As in prior work, we conducted all computation for this work on a virtual installation of Windows Server 2012 R2, with an eight processor Intel Xeon 8160 CPU, and 16 GB of RAM. We had access to a shared GPU through a NVIDIA GRID V100D-16Q, with 16 GB of VRAM allocated to our virtualisation. We ran the neural models on the virtual GPU, and BoW models on the CPU.

Implementation

As done in the prior work, we implemented our BoW model with the scikit-learn library[12], while we used PyTorch[13] and PyTorch Lightning[14] for our neural models, the last being used to reduce boilerplate code. We used the Pandas data processing library[15] for data processing, target generation, and analysis. We used the Captum interpretability library to implement integrated gradients[16].

We used loss weighting to account for the class imbalance of our targets, as most patients did not see a psychiatrist or counsellor in the first twelve months. We adjusted our binary cross entropy loss by the inverse of the relative class proportion.

Once published, code is available on the public GitHub repository https://github.com/jjnunez11/scar_nlp_psych.

Novel Multiple Document Neural Interpretation Technique

To interpret a CNN model that predicted seeing a psychiatrist, and a model predicting seeing a counsellor, we first extracted sentences from the documents in the test set. We extracted sentences that had a mean positive attribution score of at least 0.01 after being scored using Captum’s layered integrated gradients. During this process, documents were truncated to a maximum of 1500 tokens due to computational constraints. We then provided the sentences to a BERTopic topic model[17]. We generally used the parameters suggested as best practice by the author as of August 28, 2023. We did not specify a sentence transformer, so the default *all-MiniLM-L6-v2* model was used. We used UMAP[18] with *n_neighbors*=15, *n_components*=5, *min_dist*=0.0, *metric*=’cosine’ and fixed *random_state* to 42 to keep results consistent. We used HBDSCAN[19] with *min_cluster_size*=150, *metric*=’euclidean’, *cluster_selection*=’eom’, *prediction_data*=True. We used a scikit-learn[12] *CountVectorizer* with *stop_words*=’english’, *min_df*=2, *ngram_range*=(1, 2). We utilized *nr_topics*=21 so that we would have 20 topics,

discarding the “-1” topic which represents outliers. For representations, we added KeyBERT with default parameters[20], and used OpenAI’s ChatGPT[21]. Using ChatGPT will send the top four representative sentences for each topic to OpenAI, which we manually checked to ensure did not have any patient information. While OpenAI states they do not store data sent through their API, we require an extra flag and check for OpenAI to be used in our code to ensure there is no accidental data leakage. Please see the README for more details.

We used ChatGPT with *model*=’gpt-3.5-tubro’, *exponential_backoff*=True, *chat*=True, and we used the following prompt:

```
"""   I have a topic that contains the following documents:
      [DOCUMENTS]
      The topic is described by the following keywords: [KEYWORDS]

      Based on the information above, extract a short but highly descriptive topic label of at most 5 words. Make
      sure it is in the following format: topic: <topic label> """
```

Note SN2. Visualizing word importance of CNN models used to which patients see a counsellor or psychiatrist in the 12 months following initial oncologist consultation document generation

Seeing a psychiatrist

This single 41 year old woman was referred to us today for adjunctive chemotherapy due to a large pelvic mass. History of Presenting Illness. Her history started only a few months ago. She had ultrasound done in the context of reporting irregular menstrual cycles and some occasional spotting to her nurse practitioner. Ultrasound last month showed a right adnexal lesions now 8. 1 x 8 . 0 x 9 . 5 with multiple peripheral nodules. The largest of these peripheral nodules were 4.8 cm. cal5 3 was slightly elevated, other markers were normal. Today in the clinic, she is reporting pain in the lower abdomen, localizing to the right and sometimes making it hard to walk; she is not sure when this started, but suspects it has been at least a few weeks. She has also noticed increasing lose stools over this time, as well as increased flatulence and some urinary frequency. Fortunately, her appetite is intact, and she has not lost any weight. Gynecological History her menarche was at age 12. She reports that her menstrual cycles are usually quite heavy, and typically last 28 days. She has no history of sexual transmitted infections, and she is currently sexually active. Her last mammogram was about six months ago. Past medical and surgical history she is otherwise health, and reports no prior operations. Medications she takes none. allergies no known to drugs. Family History her mother has no history of cancer, though her maternal grandmother had breast at age 42. A maternal uncle had leukemia as a child. Personal history she grew up here in Vancouver and is currently in a relationship, but they lives alone. She denies tobacco or alcohol use, and uses cannabis about once per week. She is currently able to work at her job in retail. Physical Exam She appears younger than her stated age. Her weight was 164 cm, with a weight of 62 kg, and vital signs were normal, with a blood pressure of 108 64 and a heart rate of 71 beats per minute. Her lungs were clear, and I could not find any lymphadenopathy.

Seeing a counsellor

This single 41 year old woman was referred to us today for adjunctive chemotherapy due to a large pelvic mass. History of Presenting Illness. Her history started only a few months ago. She had ultrasound done in the context of reporting irregular menstrual cycles and some occasional spotting to her nurse practitioner. Ultrasound last month showed a right adnexal lesions now 8. 1 x 8 . 0 x 9 . 5 with multiple peripheral nodules. The largest of these peripheral nodules were 4.8 cm. cal5 3 was slightly elevated, other markers were normal. Today in the clinic, she is reporting pain in the lower abdomen, localizing to the right and sometimes making it hard to walk; she is not sure when this started, but suspects it has been at least a few weeks. She has also noticed increasing lose stools over this time, as well as increased flatulence and some urinary frequency. Fortunately, her appetite is intact, and she has not lost any weight. Gynecological History her menarche was at age 12. She reports that her menstrual cycles are usually quite heavy, and typically last 28 days. She has no history of sexual transmitted infections, and she is currently sexually active. Her last mammogram was about six months ago. Past medical and surgical history she is otherwise health, and reports no prior operations. Medications she takes none. allergies no known to drugs. Family History her mother has no history of cancer, though her maternal grandmother had breast at age 42. A maternal uncle had leukemia as a child. Personal history she grew up here in Vancouver and is currently in a relationship, but they lives alone. She denies tobacco or alcohol use, and uses cannabis about once per week. She is currently able to work at her job in retail. Physical Exam She appears younger than her stated age. Her weight was 164 cm, with a weight of 62 kg, and vital signs were normal, with a blood pressure of 108 64 and a heart rate of 71 beats per minute. Her lungs were clear, and I could not find any lymphadenopathy.

We show a visualization of word importance using integrated gradients for convolutional neural network models predicting whether patients will see a psychiatrist or counsellor in the 12 months following their initial oncologist consultation document being generated. We show a synthesized document created to have similar word importance to a document from a patient that saw both a counsellor and psychiatrist. The darker the green background of a token the more it predicted seeing the provider in this context, while the redder, the more it was a negative predictor. These are the default Captum colors. The modes predict that this patient will see both providers. CNN, convolutional neural network.

Note SN2. Visualizing word importance of CNN models used to which patients see a counsellor or psychiatrist in the 12 months following initial oncologist consultation document generation, adapter for colour blindness

Seeing a psychiatrist

This single 41 year old woman was referred to us today for adjunctive chemotherapy due to a large pelvic mass.. History of Presenting Illness. Her history started only a few months ago. She had ultrasound done in the context of reporting irregular menstrual cycles and some occasional spotting to her nurse practitioner. Ultrasound last month showed a right adnexal lesions now 8. 1 x 8 . 0 x 9 . 5 with multiple peripheral nodules. The largest of these peripheral nodules were 4.8 cm. cal5 3 was slightly elevated, other markers were normal. Today in the clinic, she is reporting pain in the lower abdomen, localizing to the right and sometimes making it hard to walk; she is not sure when this started, but suspects it has been at least a few weeks. She has also noticed increasing lose stools over this time, as well as increased flatulence and some urinary frequency. Fortunately, her appetite is intact, and she has not lost any weight. Gynecological History her menarche was at age 12. She reports that her menstrual cycles are usually quite heavy, and typically last 28 days. She has no history of sexual transmitted infections, and she is currently sexually active. Her last mammogram was about six months ago. Past medical and surgical history she is otherwise health, and reports no prior operations. Medications she takes none. allergies no known to drugs. Family History her mother has no history of cancer, though her maternal grandmother had breast at age 42. A maternal uncle had leukemia as a child. Personal history she grew up here in Vancouver and is currently in a relationship, but they lives alone. She denies tobacco or alcohol use, and uses cannabis about once per week. She is currently able to work at her job in retail. Physical Exam She appears younger than her stated age. Her weight was 164 cm, with a weight of 62 kg, and vital signs were normal, with a blood pressure of 108 64 and a heart rate of 72 beats per minute. Her lungs were clear, and I could not find any lymphadenopathy.

Seeing a counsellor

This single 41 year old woman was referred to us today for adjunctive chemotherapy due to a large pelvic mass. History of Presenting Illness. Her history started only a few months ago. She had ultrasound done in the context of reporting irregular menstrual cycles and some occasional spotting to her nurse practitioner. Ultrasound last month showed a right adnexal lesions now 8. 1 x 8 . 0 x 9 . 5 with multiple peripheral nodules. The largest of these peripheral nodules were 4.8 cm. cal5 3 was slightly elevated, other markers were normal. Today in the clinic, she is reporting pain in the lower abdomen, localizing to the right and sometimes making it hard to walk; she is not sure when this started, but suspects it has been at least a few weeks. She has also noticed increasing lose stools over this time, as well as increased flatulence and some urinary frequency. Fortunately, her appetite is intact, and she has not lost any weight. Gynecological History her menarche was at age 12. She reports that her menstrual cycles are usually quite heavy, and typically last 28 days. She has no history of sexual transmitted infections, and she is currently sexually active. Her last mammogram was about six months ago. Past medical and surgical history she is otherwise health, and reports no prior operations. Medications she takes none. allergies no known to drugs. Family History her mother has no history of cancer, though her maternal grandmother had breast at age 42. A maternal uncle had leukemia as a child. Personal history she grew up here in Vancouver and is currently in a relationship, but they lives alone. She denies tobacco or alcohol use, and uses cannabis about once per week. She is currently able to work at her job in retail. Physical Exam She appears younger than her stated age. Her weight was 164 cm, with a weight of 62 kg, and vital signs were normal, with a blood pressure of 108 64 and a heart rate of 71 beats per minute. Her lungs were clear, and I could not find any lymphadenopathy.

We show a visualization of word importance using integrated gradients for convolutional neural network models predicting whether patients will see a psychiatrist or counsellor in the 12 months following their initial oncologist consultation document being generated. We show a synthesized document created to have similar word importance to a document from a patient that saw both a counsellor and psychiatrist. The darker the green background of a token the more it predicted seeing the provider in this context, while the more purple, the more it was a negative predictor. The modes predict that this patient will see both providers. CNN, convolutional neural network.

SR1: Supplementary References

1. Nunez, J.-J., Leung, B., Ho, C., Bates, A. T. & Ng, R. T. Predicting the Survival of Patients With Cancer From Their Initial Oncology Consultation Document Using Natural Language Processing. *JAMA Network Open* **6**, e230813 (2023).
2. Zhang, A., Lipton, Z. C., Li, M. & Smola, A. J. Dive into deep learning. *arXiv preprint arXiv:2106.11342* (2021).
3. Manning, C., Raghavan, P. & Schuetze, H. *Introduction to Information Retrieval*. (Cambridge University Press, 2009).
4. Kim, Y. Convolutional Neural Networks for Sentence Classification. in *Proceedings of the 2014 Conference on Empirical Methods in Natural Language Processing (EMNLP)* 1746–1751 (Association for Computational Linguistics, 2014). doi:10.3115/v1/D14-1181.
5. Rios, A. & Kavuluru, R. Convolutional neural networks for biomedical text classification: application in indexing biomedical articles. in *Proceedings of the 6th ACM Conference on Bioinformatics, Computational Biology and Health Informatics* 258–267 (ACM, 2015). doi:10.1145/2808719.2808746.
6. Rios, A. & Kavuluru, R. Ordinal Convolutional Neural Networks for Predicting RDoC Positive Valence Psychiatric Symptom Severity Scores. *J Biomed Inform* **75 Suppl**, S85–S93 (2017).
7. Adhikari, A., Ram, A., Tang, R. & Lin, J. Rethinking Complex Neural Network Architectures for Document Classification. in *Proceedings of the 2019 Conference of the North* 4046–4051 (Association for Computational Linguistics, 2019). doi:10.18653/v1/N19-1408.
8. Devlin, J., Chang, M.-W., Lee, K. & Toutanova, K. BERT: Pre-training of Deep Bidirectional Transformers for Language Understanding. *arXiv:1810.04805 [cs]* (2019).
9. Huang, K., Altosaar, J. & Ranganath, R. ClinicalBERT: Modeling Clinical Notes and Predicting Hospital Readmission. *arXiv:1904.05342 [cs]* (2019).
10. Lee, J. *et al.* BioBERT: a pre-trained biomedical language representation model for biomedical text mining. *Bioinformatics* **36**, 1234–1240 (2020).
11. Beltagy, I., Peters, M. E. & Cohan, A. Longformer: The Long-Document Transformer. *arXiv:2004.05150 [cs]* (2020).
12. Pedregosa, F. *et al.* Scikit-learn: Machine Learning in Python. *Journal of Machine Learning Research* **12**, 2825–2830 (2011).

13. Paszke, A. *et al.* PyTorch: An Imperative Style, High-Performance Deep Learning Library. in *Advances in Neural Information Processing Systems* vol. 32 (Curran Associates, Inc., 2019).
14. Falcon, W. *et al.* PyTorchLightning/pytorch-lightning: 0.7.6 release. (2020) doi:10.5281/ZENODO.3828935.
15. McKinney, W. & others. pandas: a foundational Python library for data analysis and statistics. *Python for high performance and scientific computing* **14**, 1–9 (2011).
16. Kokhlikyan, N. *et al.* Captum: A unified and generic model interpretability library for PyTorch. *arXiv:2009.07896 [cs, stat]* (2020).
17. Grootendorst, M. BERTopic: Neural topic modeling with a class-based TF-IDF procedure. Preprint at https://doi.org/10.48550/arXiv.2203.05794 (2022).
18. McInnes, L., Healy, J. & Melville, J. UMAP: Uniform Manifold Approximation and Projection for Dimension Reduction. Preprint at https://doi.org/10.48550/arXiv.1802.03426 (2020).
19. McInnes, L., Healy, J. & Astels, S. hdbscan: Hierarchical density based clustering. *Journal of Open Source Software* **2**, 205 (2017).
20. Grootendorst, M. KeyBERT: Minimal keyword extraction with BERT. (2020) doi:10.5281/zenodo.4461265.
21. OpenAI. ChatGPT (Version 3.5 Turbo). (2023).

Supplementary Tables

Table ST1: Definition of evaluation metrics reported in this work

Metric	Definition
Accuracy	$\frac{TP+TN}{TP+TN+FP+FN}$
Balanced Accuracy	$\frac{\frac{TP}{TP+FN}+\frac{TN}{TN+FP}}{2}$
Recall/Sensitivity	$\frac{TP}{TP+FN}$
Specificity	$\frac{TN}{TN+FP}$
Precision/Positive Predictive Value	$\frac{TP}{TP+FP}$
Negative Predictive Value	$\frac{TN}{TN+FN}$
F1	$\frac{2TP}{2TP+FP+FN}$
AUC	Integral of the receiver-operator-curve, formed by plotting sensitivity against 1-specificity at various detection thresholds.

TP, true positive; TF, true negative; FP, false positive; FN, false negative; AUC, receiver-operator curve area-under-curve.

Table ST2. Performance of all models when predicting if patients will see a psychiatrist in twelve months, with extended metrics

Model	Acc.	BAC	AUC	F1	Rec.	Prec.	Spec.	PPV	NPV	TP	TN	FP	FN
Rule[a]	0.981309	0.5416	0.541586	0.126829	0.087838	0.228070	0.995334	0.228070	0.985819	13	9385	44	135
BoW	0.876788	0.6847	0.783699	0.108761	0.486486	0.061224	0.882914	0.061224	0.990953	72	8325	1104	76
BoW	0.876788	0.6847	0.783699	0.108761	0.486486	0.061224	0.882914	0.061224	0.990953	72	8325	1104	76
BoW	0.876788	0.6847	0.783699	0.108761	0.486486	0.061224	0.882914	0.061224	0.990953	72	8325	1104	76
BoW	0.876788	0.6847	0.783699	0.108761	0.486486	0.061224	0.882914	0.061224	0.990953	72	8325	1104	76
BoW	0.876788	0.6847	0.783699	0.108761	0.486486	0.061224	0.882914	0.061224	0.990953	72	8325	1104	76
BoW	0.876788	0.6847	0.783699	0.108761	0.486486	0.061224	0.882914	0.061224	0.990953	72	8325	1104	76
BoW	0.876788	0.6847	0.783699	0.108761	0.486486	0.061224	0.882914	0.061224	0.990953	72	8325	1104	76
BoW	0.876788	0.6847	0.783699	0.108761	0.486486	0.061224	0.882914	0.061224	0.990953	72	8325	1104	76
BoW	0.876788	0.6847	0.783699	0.108761	0.486486	0.061224	0.882914	0.061224	0.990953	72	8325	1104	76
BoW	0.876788	0.6847	0.783699	0.108761	0.486486	0.061224	0.882914	0.061224	0.990953	72	8325	1104	76
CNN	0.883471	0.737975	0.831328	0.134884	0.587838	0.076182	0.888111	0.076182	0.992768	87	8374	1055	61
CNN	0.883262	0.697964	0.787865	0.118297	0.506757	0.066964	0.889172	0.066964	0.991368	75	8384	1045	73
CNN	0.834917	0.74657	0.831964	0.109296	0.655405	0.059619	0.837735	0.059619	0.993585	97	7899	1530	51
CNN	0.82103	0.706264	0.797858	0.092161	0.587838	0.05	0.82469	0.05	0.992216	87	7776	1653	61
CNN	0.868957	0.733929	0.828892	0.122991	0.594595	0.068589	0.873263	0.068589	0.992766	88	8234	1195	60
CNN	0.827399	0.746077	0.832658	0.106003	0.662162	0.057613	0.829993	0.057613	0.993652	98	7826	1603	50
CNN	0.85225	0.738746	0.830845	0.115072	0.621622	0.063405	0.85587	0.063405	0.993109	92	8070	1359	56
CNN	0.87731	0.72487	0.829692	0.125093	0.567568	0.070293	0.882172	0.070293	0.992365	84	8318	1111	64
CNN	0.810588	0.73754	0.833404	0.097512	0.662162	0.052632	0.812918	0.052632	0.993519	98	7665	1764	50
CNN	0.85131	0.741594	0.835222	0.115528	0.628378	0.063611	0.85481	0.063611	0.993222	93	8060	1369	55
LSTM	0.747416	0.72541	0.799206	0.079178	0.702703	0.041952	0.748118	0.041952	0.993801	104	7054	2375	44
LSTM	0.746789	0.725092	0.785034	0.078997	0.702703	0.041851	0.747481	0.041851	0.993796	104	7048	2381	44
LSTM	0.798162	0.73788	0.807552	0.093765	0.675676	0.050378	0.800085	0.050378	0.993678	100	7544	1885	48
LSTM	0.79503	0.713012	0.806033	0.086552	0.628378	0.046477	0.797646	0.046477	0.99274	93	7521	1908	55
LSTM	0.714942	0.735521	0.803156	0.075829	0.756757	0.039914	0.714286	0.039914	0.994683	112	6735	2694	36
LSTM	0.756918	0.733561	0.807713	0.082742	0.709459	0.043933	0.757663	0.043933	0.994017	105	7144	2285	43
LSTM	0.76005	0.711874	0.786058	0.078589	0.662162	0.041773	0.761587	0.041773	0.993085	98	7181	2248	50
LSTM	0.813512	0.709097	0.788227	0.090631	0.601351	0.049009	0.816842	0.049009	0.992398	89	7702	1727	59
LSTM	0.837214	0.737761	0.808362	0.107613	0.635135	0.058787	0.840386	0.058787	0.993231	94	7924	1505	54
LSTM	0.852877	0.712461	0.798475	0.106531	0.567568	0.058782	0.857355	0.058782	0.992145	84	8084	1345	64
BERT	0.953117	0.550546	0.685491	0.0818	0.135135	0.058651	0.965956	0.058651	0.986141	20	9108	321	128
BERT	0.869583	0.674391	0.737237	0.100792	0.472973	0.056406	0.875809	0.056406	0.990643	70	8258	1171	78
BERT	0.89527	0.617603	0.715341	0.08901	0.331081	0.051417	0.904126	0.051417	0.98852	49	8525	904	99
BERT	0.863527	0.631411	0.708066	0.081518	0.391892	0.04549	0.87093	0.04549	0.989159	58	8212	1217	90
BERT	0.888901	0.604393	0.707318	0.079585	0.310811	0.045635	0.897974	0.045635	0.988097	46	8467	962	102
BERT	0.850057	0.6678	0.732892	0.089987	0.47973	0.04965	0.85587	0.04965	0.990549	71	8070	1359	77
BERT	0.924611	0.599251	0.709678	0.0975	0.263514	0.059816	0.934988	0.059816	0.987787	39	8816	613	109
BERT	0.939752	0.573686	0.709092	0.091339	0.195946	0.059548	0.951426	0.059548	0.986909	29	8971	458	119
BERT	0.958964	0.536888	0.637991	0.070922	0.101351	0.054545	0.972426	0.054545	0.985702	15	9169	260	133
BERT	0.857575	0.64169	0.723286	0.083333	0.418919	0.046269	0.864461	0.046269	0.989559	62	8151	1278	86

Acc.: accuracy, BAC: balanced accuracy, AUC: receiver-operator-curve area-under-curve, Rec.: Recall/Sensitivity, Prec.: Precision, Spec.: Specificity, PPV: positive predictive value, NPV: negative predictive value, TP: true positives, TN: true negatives, FP: false positives, FN: false negatives, BoW: bag-of-words, CNN: convolutional neural networks, LSTM: long short-term memory, BERT: bidirectional encoder representations from transformers.

[a] Rule-based method that predicts a patient will see a psychiatrist if the document contains the token “psychiatrist”.

Table ST3. Statistical comparison of models when predicting if patients will see a psychiatrist in the twelve months following their initial oncologist consultation

**Balanced Accuracy**					
**Model**	**BoW**	**CNN**	**LSTM**	**BERT**	**Rule**
**BoW**	NA	0.000010	0.000002	0.000636	0
**CNN**	3.96	NA	0.22	0.000054	0
**LSTM**	4.77	-0.49	NA	0.000039	0
**BERT**	-2.29	-3.49	-3.38	NA	0.001205
**Rule**	Inf	11.41	15.60	1.47	NA
**AUC**					
**Model**	**BoW**	**CNN**	**LSTM**	**BERT**	**Rule**
**BoW**	NA	0.000032	0.00058	0.000012	0
**CNN**	3.42	NA	0.00098	0.000004	0
**LSTM**	2.32	-1.85	NA	0.000009	0
**BERT**	-3.86	-5.07	-4.4	NA	0
**Rule**	Inf	16.95	27.61	5.885	NA

P-values from running two-tailed dependent t-tests between ten runs of each model are shaded light grey, in the top-right of each grid. Cohen's d are shown in the bottom-left of each grid. For the t-tests, using Bonferroni correction, we adjust alpha at 95% confidence to 0.05/6. AUC: receiver-operator-curve area-under-curve, BAC: balanced accuracy, BERT: bidirectional encoder representations from transformers, CNN: convolutional neural networks, LSTM: long short-term memory.

Table ST4. Performance of all models when predicting if patients will see a counsellor in twelve months, with extended metrics

Model	Acc.	BAC	AUC	F1	Rec.	Prec.	Spec.	PPV	NPV	TP	TN	FP	FN
Rule[a]	0.784379	0.553216	0.553216	0.229765	0.151500	0.475309	0.954931	0.475309	0.806809	308	7204	340	1725
BoW	0.705336	0.696721	0.764259	0.495531	0.681751	0.389217	0.711691	0.389217	0.892453	1386	5369	2175	647
BoW	0.705336	0.696721	0.764259	0.495531	0.681751	0.389217	0.711691	0.389217	0.892453	1386	5369	2175	647
BoW	0.705336	0.696721	0.764259	0.495531	0.681751	0.389217	0.711691	0.389217	0.892453	1386	5369	2175	647
BoW	0.705336	0.696721	0.764259	0.495531	0.681751	0.389217	0.711691	0.389217	0.892453	1386	5369	2175	647
BoW	0.705336	0.696721	0.764259	0.495531	0.681751	0.389217	0.711691	0.389217	0.892453	1386	5369	2175	647
BoW	0.705336	0.696721	0.764259	0.495531	0.681751	0.389217	0.711691	0.389217	0.892453	1386	5369	2175	647
BoW	0.705336	0.696721	0.764259	0.495531	0.681751	0.389217	0.711691	0.389217	0.892453	1386	5369	2175	647
BoW	0.705336	0.696721	0.764259	0.495531	0.681751	0.389217	0.711691	0.389217	0.892453	1386	5369	2175	647
BoW	0.705336	0.696721	0.764259	0.495531	0.681751	0.389217	0.711691	0.389217	0.892453	1386	5369	2175	647
BoW	0.705336	0.696721	0.764259	0.495531	0.681751	0.389217	0.711691	0.389217	0.892453	1386	5369	2175	647
CNN	0.717866	0.71761	0.784566	0.519046	0.717167	0.406695	0.718054	0.406695	0.904039	1458	5417	2127	575
CNN	0.759632	0.706572	0.786406	0.520417	0.614363	0.451391	0.798781	0.451391	0.884875	1249	6026	1518	784
CNN	0.753159	0.708751	0.783587	0.520681	0.631579	0.442911	0.785923	0.442911	0.887841	1284	5929	1615	749
CNN	0.724235	0.713928	0.783135	0.517273	0.696016	0.411577	0.73184	0.411576	0.899332	1415	5521	2023	618
CNN	0.714107	0.714146	0.78406	0.514711	0.714215	0.402328	0.714077	0.402328	0.902647	1452	5387	2157	581
CNN	0.721416	0.71142	0.781593	0.514026	0.694048	0.408157	0.728791	0.408157	0.898366	1411	5498	2046	622
CNN	0.760885	0.700899	0.783771	0.514419	0.596655	0.452106	0.805143	0.452106	0.881056	1213	6074	1470	820
CNN	0.733633	0.710371	0.782874	0.516398	0.669946	0.420111	0.750795	0.420111	0.894081	1362	5664	1880	671
CNN	0.757962	0.708206	0.785929	0.521667	0.621741	0.449342	0.794671	0.449342	0.88631	1264	5995	1549	769
CNN	0.674637	0.713168	0.783738	0.504453	0.780128	0.372738	0.646209	0.372738	0.916009	1586	4875	2669	447
LSTM	0.698757	0.711051	0.782175	0.507931	0.732415	0.388773	0.689687	0.388773	0.905342	1489	5203	2341	544
LSTM	0.62222	0.697863	0.779613	0.482403	0.829316	0.340125	0.56641	0.340125	0.924892	1686	4273	3271	347
LSTM	0.731544	0.699164	0.776183	0.504147	0.642892	0.414657	0.755435	0.414657	0.887004	1307	5699	1845	726
LSTM	0.749608	0.705419	0.780897	0.515947	0.628628	0.437521	0.782211	0.437521	0.886569	1278	5901	1643	755
LSTM	0.752428	0.700921	0.776711	0.511839	0.611412	0.440156	0.790429	0.440156	0.883015	1243	5963	1581	790
LSTM	0.70544	0.708286	0.781321	0.506904	0.713232	0.393167	0.70334	0.393167	0.901002	1450	5306	2238	583
LSTM	0.725384	0.709986	0.778894	0.513683	0.683227	0.411556	0.736744	0.411556	0.896163	1389	5558	1986	644
LSTM	0.739689	0.712418	0.785851	0.5203	0.665027	0.427307	0.759809	0.427307	0.893809	1352	5732	1812	681
LSTM	0.726428	0.710109	0.782191	0.514095	0.681751	0.412623	0.738468	0.412623	0.895947	1386	5571	1973	647
LSTM	0.712227	0.705587	0.779552	0.505916	0.694048	0.398025	0.717126	0.398025	0.896883	1411	5410	2134	622
BERT	0.696356	0.635685	0.704676	0.42575	0.530251	0.355658	0.741119	0.355658	0.854109	1078	5591	1953	955
BERT	0.598413	0.656701	0.720701	0.444861	0.757993	0.314811	0.555408	0.314811	0.894917	1541	4190	3354	492
BERT	0.726428	0.639501	0.710981	0.431177	0.488441	0.385931	0.790562	0.385931	0.851513	993	5964	1580	1040
BERT	0.705753	0.638057	0.707937	0.428861	0.520413	0.364702	0.7557	0.364702	0.853954	1058	5701	1843	975
BERT	0.680589	0.644362	0.707377	0.435921	0.581407	0.348673	0.707317	0.348673	0.862454	1182	5336	2208	851
BERT	0.660123	0.64808	0.706056	0.439276	0.627152	0.338017	0.669008	0.338017	0.869423	1275	5047	2497	758
BERT	0.707528	0.640082	0.711944	0.4315	0.522873	0.367312	0.757291	0.367312	0.854856	1063	5713	1831	970
BERT	0.626501	0.654946	0.716736	0.444651	0.704378	0.324864	0.605514	0.324864	0.88373	1432	4568	2976	601
BERT	0.732066	0.526478	0.551721	0.211432	0.169208	0.281736	0.883749	0.281736	0.79787	344	6667	877	1689
BERT	0.694372	0.525549	0.555749	0.243865	0.232169	0.256801	0.818929	0.256801	0.798294	472	6178	1366	1561

Acc.: accuracy, BAC: balanced accuracy, AUC: receiver-operator-curve area-under-curve, Rec.: Recall/Sensitivity, Prec.: Precision, Spec.: Specificity, PPV: positive predictive value, NPV: negative predictive value, TP: true positives, TN: true negatives, FP: false positives, FN: false negatives, BoW: bag-of-words, CNN: convolutional neural networks, LSTM: long short-term memory, BERT: bidirectional encoder representations from transformers.

[a] Rule-based method that predicts a patient will see a psychiatrist if the document contains the token "psychiatrist".

Table ST5. Statistical comparison of models when predicting if patients will see a counselor in the twelve months following their initial oncologist consultation

**Balanced Accuracy**					
**Model**	**BoW**	**CNN**	**LSTM**	**BERT**	**Rule**
BoW	NA	0.000007	0.00030	0.00105	0
CNN	4.14	NA	0.07066	0.00036	0
LSTM	2.54	-0.89	NA	0.00057	0
BERT	-2.12	-2.5	-2.37	NA	0.002171
**Rule**	Inf	33.41	29.37	1.34	NA
**AUC**					
**Model**	**BoW**	**CNN**	**LSTM**	**BERT**	**Rule**
**BoW**	NA	0	0	0.0029	0
**CNN**	19.75	NA	0.0071	0.00079	0
**LSTM**	8.03	-1.62	NA	0.0010	0
**BERT**	-1.81	-2.23	-2.15	NA	0.0002
**Rule**	Inf	163.53	80.23	1.90	NA

P-values from running two-tailed dependent t-tests between ten runs of each model are shaded light grey, in the top-right of each grid. Cohen's d are shown in the bottom-left of each grid. For the t-tests, using Bonferroni correction, we adjust alpha at 95% confidence to 0.05/6. Values less than 0.000001 are marked 0. AUC: receiver-operator-curve area-under-curve, BAC: balanced accuracy, BERT: bidirectional encoder representations from transformers, CNN: convolutional neural networks, LSTM: long short-term memory.

Table ST6. Statistical comparison of each model when predicting if patients will see a psychiatrist versus seeing a counselor, in the twelve months following their initial oncologist consultation

**BAC**	P-value	Cohen's d
BoW	0	Inf
CNN	0.0040	1.69
LSTM	0.0030	2.0
BERT	0.56	-0.23
**AUC**		
BoW	0	Inf
CNN	0	3.38
LSTM	0	2.71
BERT	0.17	0.53

P-values from running two-tailed dependent t-tests between ten runs of each model. Using Bonferroni correction, we adjust alpha at 95% confidence to 0.05/4. Values less than 0.000001 are marked 0. We also show Cohen's d effect sizes. Abbreviations**:** AUC: receiver-operator-curve area-under-curve, BAC: balanced accuracy, BERT: bidirectional encoder representations from transformers, CNN: convolutional neural networks, LSTM: long short-term memory.

Table ST7. Performance Longformer, CNN, and BERT models when predicting if patients will see a psychiatrist with different numbers of maximum tokens, with extended metrics

Model	Max Tokens	Batch Size	Accuracy	Balanced Accuracy	AUC	F1	Recall	Precision	Specificity	PPV	NPV	TP	TN	FP	FN
Longformer	512	8	0.844106	0.604921	0.689829	0.066291	0.358108	0.036527	0.851734	0.036527	0.988309	53	8031	1398	95
Longformer	512	8	0.767255	0.649026	0.703784	0.065409	0.527027	0.034868	0.771026	0.034868	0.990463	78	7270	2159	70
Longformer	512	8	0.426438	0.622259	0.686116	0.042531	0.824324	0.021829	0.420193	0.021829	0.99348	122	3962	5467	26
Longformer	512	8	0.727368	0.655372	0.706131	0.061804	0.581081	0.032638	0.729664	0.032638	0.991069	86	6880	2549	62
Longformer	512	8	0.630573	0.642794	0.70517	0.051983	0.655405	0.027065	0.630183	0.027065	0.99149	97	5942	3487	51
Longformer	512	8	0.938498	0.586351	0.730474	0.100763	0.222973	0.065089	0.94973	0.065089	0.987321	33	8955	474	115
Longformer	512	8	0.919286	0.616498	0.723589	0.104287	0.304054	0.062937	0.928943	0.062937	0.988377	45	8759	670	103
Longformer	512	8	0.774146	0.615947	0.686146	0.058337	0.452703	0.031177	0.779192	0.031177	0.989095	67	7347	2082	81
Longformer	512	8	0.774146	0.672478	0.742223	0.072072	0.567568	0.038479	0.777389	0.038479	0.991344	84	7330	2099	64
Longformer	512	8	0.879921	0.633086	0.723057	0.088748	0.378378	0.050269	0.887793	0.050269	0.989129	56	8371	1058	92
Longformer	1024	4	0.82907	0.610586	0.650448	0.065106	0.385135	0.035558	0.836038	0.035558	0.988588	57	7883	1546	91
Longformer	1024	4	0.636421	0.639113	0.69632	0.051743	0.641892	0.026958	0.636335	0.026958	0.991244	95	6000	3429	53
Longformer	1024	4	0.74491	0.694209	0.764738	0.072161	0.641892	0.038229	0.746527	0.038229	0.992527	95	7039	2390	53
Longformer	1024	4	0.905294	0.649297	0.754929	0.111655	0.385135	0.065292	0.913458	0.065292	0.989545	57	8613	816	91
Longformer	1024	4	0.812154	0.688455	0.763042	0.084478	0.560811	0.04568	0.816099	0.04568	0.991624	83	7695	1734	65
Longformer	1024	4	0.853816	0.619829	0.719196	0.074074	0.378378	0.041056	0.861279	0.041056	0.988798	56	8121	1308	92
Longformer	1024	4	0.684452	0.650205	0.713488	0.056804	0.614865	0.029777	0.685545	0.029777	0.991259	91	6464	2965	57
Longformer	1024	4	0.842957	0.667519	0.757155	0.087379	0.486486	0.048	0.848552	0.048	0.990591	72	8001	1428	76
Longformer	1024	4	0.621071	0.671222	0.714134	0.055686	0.722973	0.028958	0.619472	0.028958	0.99303	107	5841	3588	41
Longformer	1024	4	0.939647	0.560332	0.697201	0.079618	0.168919	0.052083	0.951745	0.052083	0.986479	25	8974	455	123
Longformer	2048	2	0.768821	0.676425	0.733453	0.072087	0.581081	0.038427	0.771768	0.038427	0.991552	86	7277	2152	62
Longformer	2048	2	0.735721	0.672916	0.73758	0.066396	0.608108	0.035115	0.737724	0.035115	0.991731	90	6956	2473	58
Longformer	2048	2	0.753472	0.661979	0.735238	0.066429	0.567568	0.035279	0.75639	0.035279	0.991106	84	7132	2297	64
Longformer	2048	2	0.722564	0.66956	0.72138	0.064107	0.614865	0.033816	0.724255	0.033816	0.991722	91	6829	2600	57
Longformer	2048	2	0.816853	0.67754	0.744134	0.082636	0.533784	0.044785	0.821296	0.044785	0.991169	79	7744	1685	69
Longformer	2048	2	0.776339	0.716821	0.768859	0.083048	0.655405	0.044333	0.778237	0.044333	0.993098	97	7338	2091	51
Longformer	2048	2	0.758275	0.667743	0.730124	0.06841	0.574324	0.036371	0.761162	0.036371	0.991298	85	7177	2252	63
Longformer	2048	2	0.837005	0.607966	0.709367	0.065829	0.371622	0.036113	0.84431	0.036113	0.988453	55	7961	1468	93
Longformer	2048	2	0.851624	0.661945	0.750624	0.088518	0.466216	0.048901	0.857673	0.048901	0.990326	69	8087	1342	79
Longformer	2048	2	0.809961	0.644112	0.714342	0.071429	0.472973	0.038631	0.815251	0.038631	0.989955	70	7687	1742	78
Longformer	4096	1	0.836274	0.614245	0.690974	0.067776	0.385135	0.037158	0.843356	0.037158	0.988686	57	7952	1477	91
Longformer	4096	1	0.663569	0.692805	0.758053	0.062282	0.722973	0.032543	0.662637	0.032543	0.993481	107	6248	3181	41
Longformer	4096	1	0.848804	0.660513	0.743963	0.087011	0.466216	0.047983	0.85481	0.047983	0.990294	69	8060	1369	79
Longformer	4096	1	0.761825	0.656245	0.710883	0.066312	0.547297	0.035294	0.765193	0.035294	0.990799	81	7215	2214	67
Longformer	4096	1	0.938812	0.589836	0.741997	0.103976	0.22973	0.067194	0.949942	0.067194	0.987432	34	8957	472	114
Longformer	4096	1	0.355957	0.606417	0.71307	0.039851	0.864865	0.020395	0.347969	0.020395	0.993941	128	3281	6148	20
Longformer	4096	1	0.686541	0.671217	0.757355	0.060701	0.655405	0.031824	0.687029	0.031824	0.992189	97	6478	2951	51
Longformer	4096	1	0.757753	0.684105	0.751367	0.072	0.608108	0.038265	0.760102	0.038265	0.991972	90	7167	2262	58
Longformer	4096	1	0.62483	0.653179	0.716149	0.053228	0.682432	0.027694	0.623926	0.027694	0.992074	101	5883	3546	47
Longformer	4096	1	0.77258	0.671683	0.75078	0.071611	0.567568	0.038217	0.775798	0.038217	0.991327	84	7315	2114	64
CNN	inf	16	0.932025	0.666197	0.799888	0.151239	0.391892	0.0937	0.940503	0.0937	0.989953	58	8868	561	90
CNN	inf	16	0.921687	0.667598	0.789531	0.137931	0.405405	0.083102	0.929791	0.083102	0.990062	60	8767	662	88

CNN	inf	16	0.889423	0.727696	0.819532	0.13551	0.560811	0.077066	0.894581	0.077066	0.992353	83	8435	994	65
CNN	inf	16	0.913438	0.690012	0.80588	0.140933	0.459459	0.083231	0.920564	0.083231	0.990868	68	8680	749	80
CNN	inf	16	0.902892	0.711259	0.804952	0.140481	0.513514	0.08137	0.909004	0.08137	0.99167	76	8571	858	72
CNN	inf	16	0.901222	0.717061	0.811936	0.141561	0.527027	0.081761	0.907095	0.081761	0.991882	78	8553	876	70
CNN	inf	16	0.926491	0.699966	0.800342	0.163895	0.466216	0.099424	0.933715	0.099424	0.991107	69	8804	625	79
CNN	inf	16	0.91584	0.691231	0.807813	0.144374	0.459459	0.085642	0.923003	0.085642	0.990891	68	8703	726	80
CNN	inf	16	0.908635	0.714175	0.811361	0.148004	0.513514	0.086462	0.914837	0.086462	0.991722	76	8626	803	72
CNN	inf	16	0.913021	0.69645	0.803805	0.143885	0.472973	0.084848	0.919928	0.084848	0.991088	70	8674	755	78
CNN	inf	1	0.852668	0.742283	0.813603	0.116468	0.628378	0.064182	0.856188	0.064182	0.993233	93	8073	1356	55
CNN	inf	1	0.882531	0.737497	0.824498	0.133949	0.587838	0.075586	0.887157	0.075586	0.992761	87	8365	1064	61
CNN	inf	1	0.8558	0.730573	0.825098	0.114176	0.601351	0.063076	0.859794	0.063076	0.992775	89	8107	1322	59
CNN	inf	1	0.870628	0.708175	0.806287	0.114367	0.540541	0.063949	0.875809	0.063949	0.991833	80	8258	1171	68
CNN	inf	1	0.86029	0.739504	0.813066	0.119737	0.614865	0.066327	0.864143	0.066327	0.993053	91	8148	1281	57
CNN	inf	1	0.910724	0.711911	0.816238	0.149254	0.506757	0.087515	0.917064	0.087515	0.991628	75	8647	782	73
CNN	inf	1	0.844523	0.738147	0.820901	0.111045	0.628378	0.060904	0.847916	0.060904	0.993168	93	7995	1434	55
CNN	inf	1	0.880234	0.736331	0.813968	0.131718	0.587838	0.074169	0.884823	0.074169	0.992742	87	8343	1086	61
CNN	inf	1	0.860604	0.719711	0.818529	0.112957	0.574324	0.062638	0.865097	0.062638	0.992336	85	8157	1272	63
CNN	inf	1	0.889318	0.714341	0.818269	0.129721	0.533784	0.073832	0.894899	0.073832	0.991889	79	8438	991	69
BERT	512	8	0.790018	0.627333	0.700556	0.063344	0.459459	0.034017	0.795206	0.034017	0.989443	68	7498	1931	80
BERT	512	8	0.816748	0.627607	0.690531	0.067977	0.432432	0.036888	0.822781	0.036888	0.989288	64	7758	1671	84
BERT	512	8	0.875326	0.600824	0.652533	0.072981	0.317568	0.041228	0.884081	0.041228	0.988029	47	8336	1093	101
BERT	512	8	0.800146	0.585922	0.650597	0.053412	0.364865	0.028815	0.806978	0.028815	0.987797	54	7609	1820	94
BERT	512	8	0.726846	0.635155	0.675578	0.057637	0.540541	0.030441	0.72977	0.030441	0.990214	80	6881	2548	68
BERT	512	8	0.812572	0.62216	0.682483	0.065591	0.425676	0.035533	0.818645	0.035533	0.989108	63	7719	1710	85
BERT	512	8	0.787512	0.61941	0.695891	0.060914	0.445946	0.032689	0.792873	0.032689	0.989151	66	7476	1953	82
BERT	512	8	0.862483	0.587651	0.662841	0.063966	0.304054	0.035743	0.871248	0.035743	0.987617	45	8215	1214	103
BERT	512	8	0.7825	0.606888	0.639132	0.057039	0.425676	0.030568	0.788101	0.030568	0.988691	63	7431	1998	85
BERT	512	8	0.711183	0.643828	0.683473	0.057902	0.574324	0.030488	0.713331	0.030488	0.99072	85	6726	2703	63
BERT	512	1	0.68581	0.640918	0.699501	0.055259	0.594595	0.028976	0.687241	0.028976	0.990826	88	6480	2949	60
BERT	512	1	0.563746	0.608857	0.671668	0.044373	0.655405	0.022964	0.562308	0.022964	0.990473	97	5302	4127	51
BERT	512	1	0.811841	0.615138	0.670369	0.06341	0.412162	0.034347	0.818114	0.034347	0.988848	61	7714	1715	87
BERT	512	1	0.547562	0.637216	0.694107	0.047483	0.72973	0.02454	0.544703	0.02454	0.992272	108	5136	4293	40
BERT	512	1	0.765375	0.614818	0.681101	0.057071	0.459459	0.030425	0.770177	0.030425	0.989104	68	7262	2167	80
BERT	512	1	0.864885	0.595522	0.677302	0.067723	0.317568	0.037903	0.873475	0.037903	0.987885	47	8236	1193	101
BERT	512	1	0.702412	0.55624	0.597668	0.040404	0.405405	0.021262	0.707074	0.021262	0.986973	60	6667	2762	88
BERT	512	1	0.945912	0.533585	0.648566	0.058182	0.108108	0.039801	0.959062	0.039801	0.985613	16	9043	386	132
BERT	512	1	0.7305	0.613734	0.645954	0.053539	0.493243	0.028306	0.734224	0.028306	0.989283	73	6923	2506	75
BERT	512	1	0.538895	0.619513	0.685575	0.044983	0.702703	0.023235	0.536324	0.023235	0.991374	104	5057	4372	44

Acc.: accuracy, BAC: balanced accuracy, AUC: receiver-operator-curve area-under-curve, Rec.: Recall/Sensitivity, Prec.: Precision, Spec.: Specificity, PPV: positive predictive value, NPV: negative predictive value, TP: true positives, TN: true negatives, FP: false positives, FN: false negatives, CNN: convolutional neural networks, BERT: bidirectional encoder representations from transformers.

Table ST8. Statistical comparison of CNN, BERT and Longformer models when predicting seeing a psychiatrist when using different numbers of tokens and undersampling.

**Balanced Accuracy**						
Model	Longformer_512	Longformer_1024	Longformer_2048	Longformer_4096	CNN	BERT_512
Longformer_512	NA	0.273986	0.024162	0.116442	0.000112	0.227184
Longformer_1024	0.45	NA	0.230655	0.782318	0.001255	0.116457
Longformer_2048	1.35	0.6	NA	0.401361	0.009786	0.000262
Longformer_4096	0.66	0.13	-0.5	NA	0.007835	0.039507
CNN	2.94	1.66	1.34	1.69	NA	0.000012
BERT_512	-0.62	-0.93	-2.1	-1.22	-4.12	NA
**AUC**						
Model	Longformer_512	Longformer_1024	Longformer_2048	Longformer_4096	CNN	BERT_512
Longformer_512	NA	0.373289	0.00249	0.042514	0	0.003935
Longformer_1024	0.45	NA	0.416968	0.392584	0.000026	0.015891
Longformer_2048	1.34	0.39	NA	0.924747	0	0.000055
Longformer_4096	1.1	0.33	-0.05	NA	0.000012	0.000126
CNN	6.41	3.07	5.19	4.08	NA	0
BERT_512	-1.79	-1.65	-3.16	-2.69	-8.3	NA

P-values from running two-tailed dependent t-tests between ten runs of each model are shaded light grey, in the top-right of each grid. Cohen's d are shown in the bottom-left of each grid. For the t-tests, using Bonferroni correction, we adjust alpha at 95% confidence to 0.05/16. Values less than 0.000001 are marked 0. AUC: receiver-operator-curve area-under-curve, BAC: balanced accuracy, BERT: bidirectional encoder representations from transformers, CNN: convolutional neural networks, LSTM: long short-term memory. Numbers following the names of models correspond to their maximum number of tokens per document; CNN has no such limit. All results compared in this table result from models being trained using undersampling, a technical requirement of us running the Longformer models.

Table ST9: Additional topic representations and example sentences from using our new interpretation technique with models trained to predict seeing a psychiatrist

Topic	Count	Maximal Marginal Relevance	KeyBERT	OpenAI	Representative Sentences
-1[a]	18804	['edema', 'scan', 'patient', 'mass', 'right', 'cm', 'referral', 'left', 'ct', 'lymph']	['peripheral edema', 'edema', 'pedal edema', 'lungs clear', 'pathology', 'bilateral', 'lymphadenopathy', 'biopsy', 'peripheral', 'diagnosis']	['Squamous cell carcinoma with no edema']	['he has no peripheral edema .', 'he had no peripheral edema .', 'there was no peripheral edema .']
0	6411	['history', 'pain', 'depression', 'past', 'discomfort', 'anxiety', 'past medical', 'medical', 'medical history', 'abdominal']	['past medical', 'history depression', 'medical history', 'depression', 'present illness', 'history past', 'illness', 'medical', 'anxiety', 'disorder']	['Medical History and Mental Health']	['past medical history delusional disorder .', 'past medical history depression .', 'past medical history depression .']
1	3606	['lymph', 'breast', 'node', 'lymph node', 'positive', 'right', 'mammogram', 'left', 'right breast', 'nodes']	['left breast', 'lymph node', 'right breast', 'lymph nodes', 'sentinel lymph', 'ductal carcinoma', 'axillary lymph', 'node biopsy', 'breast ultrasound', 'left axillary']	['Positive Breast Lymph Nodes']	['right breast is negative .', 'on 19eptember XXth , she underwent a mammogram and a sentinel lymph node biopsy which was converted to an axillary lymph node dissection due to positive nodes clinically on exam .', 'invasive ductal carcinoma of the right breast , with biopsy proven right axillary lymph node involvement , er positive (intensity 2 3 of tumor cells) , pr negative , her2 positive (3 3) .']

2	2882	[‘chemotherapy’, ‘concurrent’, ‘radiation’, ‘radiotherapy’, ‘oncology’, ‘treatment’, ‘concurrent chemotherapy’, ‘medical’, ‘medical oncology’, ‘start’]	[‘chemotherapy radiation’, ‘chemotherapy’, ‘concurrent chemotherapy’, ‘chemotherapy given’, ‘starting chemotherapy’, ‘start chemotherapy’, ‘radiation oncology’, ‘medical oncology’, ‘oncology’, ‘oncology colleagues’]	[‘Concurrent Chemotherapy and Radiation’]	[‘I also will arrange for her to be assessed by a medical oncologist with regards to the concurrent chemotherapy ’,‘’chemotherapy ’,‘’as such , she has been offered treatment with concurrent chemotherapy and radiation ’’]
3	2496	‘’c’’,‘’mas’’,‘’tumo’’,‘’showe’’,‘’lob’ ’,‘’c’’,‘’sca’’,‘’righ’’,‘’lef’’,‘’fd’’]	‘’lobe mas’’,‘’tissue mas’’,‘’tumo’’,‘’cm mas’’,‘’lower lob’’,‘’upper lob’’,‘’ct ches’’,‘’mass righ’’,‘’ct sca’’,‘’pet c’’]	‘’Lung carcinoma mass detectio’’]	‘’right hemithyroid mass ’’,‘’this showed a 3 . 2 cm , fdg avid mass within the right upper lobe , typical of primary lung carcinoma ’’,‘’the pet scan showed a right lower lobe mass measuring 1 . 6 cm in diameter that was consistent with malignant tissue and also fdg avid right hilar and subcarinal lymphadenopathy ’’]
4	2422	‘’cance’’,‘’family histor’’,‘’famil’’,‘’materna’’,‘’breast cance’’,‘’histor’’,‘’ag’’,‘’breas’’,‘’di e’’,‘’mothe’’]	‘’breast cance’’,‘’hereditary cance’’,‘’family histor’’,‘’history breas’’,‘’cousin breas’’,‘’maternal grandmothe’’,‘’maternal aun’’,‘’maternal grandfathe’’,‘’cance’’,‘’cancer diagnose’’]	‘’Breast Cancer Family Histor’’]	‘’family history his mother had breast cancer ’’,‘’family history mother had breast cancer ’’,‘’family history his mother had breast cancer ’’]
5	2039	‘’patient periphera’’,‘’ga’’,‘’patient patien’’,‘’patien’’,‘’peripheral edem’’,‘’periphera’’,‘’edem’’,‘’’,‘’’, ‘’’]	‘’’,‘’’,‘’’,‘’’,‘’’,‘’’,‘’’,‘’’,‘’’,‘’’]	‘’peripheral edem’’]	‘’r ’’,‘’r ’’,‘’there was no peripheral edema ’’]
6	1414	‘’effect’’,‘’fatigu’’,‘’nause’’,‘’includ ’’,‘’ris’’,‘’nausea vomitin’’,‘’vomitin’’,‘’neutropeni’’, ‘’effects includ’’,‘’febrile neutropeni’’]	‘’febrile neutropeni’’,‘’effects chemotherap’’,‘’alopecia nause’’,‘’myelosuppression febril’’,‘’vomiting alopeci’’,‘’risk febril’’,‘’acute effect’’,‘’fatigue nause’’,‘’neutropeni’’,‘’nause’’]	‘’Chemotherapy side effect’’]	‘’we discussed at length the side effects of chemotherapy which include but are not limited to nausea , vomiting , fatigue , myelosuppression , risk of febrile neutropenia , hair loss , mucositis , peripheral neuropathy , allergic reactions and also a small risk of dying because of chemotherapy ’’,‘’side effects include fatigue , nausea , vomiting , cytopenias with the risk of febrile neutropenia ’’,‘’side effects were discussed and may include , but are not limited to nausea , vomiting , fatigue , alopecia , mucositis , diarrhea and myelosuppression with risk of febrile neutropenia ’’]
7	1327	‘’socia’’,‘’social histor’’,‘’work’’,‘’disabilit’’,‘’live’’, ‘’toda’’,‘’wor’’,‘’history patien’’,‘’currentl’’,‘’accompanie’’]	‘’history patien’’,‘’social histor’’,‘’patient live’’,‘’patient attende’’,‘’patien’’,‘’attended clini’’,‘’socia’’,‘’hospita’’,‘’worke’’,‘’occupatio’’]	‘’Patient work histor’’]	‘’social history the patient works as a steel fabricator ’’,‘’social history the patient works at the pulp mill ’’,‘’social history the patient works as a custodian ’’]
8	1322	‘’sca’’,‘’bone sca’’,‘’bon’’,‘’pe’’,‘’c’’,‘’pet sca’’,‘’ra’’,‘’ct sca’’,‘’mr’’,‘’diseas’’]	‘’pet sca’’,‘’bone sca’’,‘’scan bon’’,‘’ct sca’’,‘’scan c’’,‘’scan performe’’,‘’mri sca’’,‘’metastatic diseas’’,‘’scan ches’’,‘’pet c’’]	‘’Metastatic disease scan’’]	‘’she had a staging ct scan and bone scan which shows no evidence of metastatic disease ’’,‘’pet scan ’’,‘’we will get a pet scan ’’]
9	1316	‘’edem’’,‘’peripheral edem’’,‘’periphera’’,‘’edema periphera’’,‘’lowe’’,‘’cal’’,‘’pittin’’, ‘’pitting edem’’,‘’edema cal’’,‘’calf tendernes’’]	‘’peripheral edem’’,‘’edema periphera’’,‘’edema bilatera’’,‘’edema bilaterall’’,‘’edema edem’’,‘’edema doe’’,‘’extremity edem’’,‘’mild edem’’,‘’edem’’,‘’significant edem’’]	‘’absence of peripheral edem’’]	‘’there is no peripheral edema ’’,‘’she has no peripheral edema ’’,‘’there is no peripheral edema ’’]
10	1160	‘’medication’’,‘’m’’,‘’curren’’,‘’curr ent medication’’,‘’medications includ’’,‘’includ’’,‘’take’’,‘’medicat ions curren’’,‘’takin’’,‘’dail’’]	‘’current medication’’,‘’medications includ’’,‘’medications currentl’’,‘’medications medication’’,‘’medications curren’’,‘’current medicatio’’,‘’medication’’,‘’mg medication’’,‘’prescriptio’’,‘’medicatio’’]	‘’Current Medication’’]	‘’medications her current medications include 1 ’’,‘’current medications ramipril 5 mg p ’’,‘’current medications include bromazepam 3 mg p ’’]

11	1111	‘'patient famil’',‘'patien’',‘'famil’',‘'counselin’',‘'family counselin’',‘'referra’',‘'discussio’',‘'understan’',‘'toda’',‘'counsellin’']	‘'family counsellin’',‘'referral patien’',‘'family counselin’',‘'referral d’',‘'counseling servic’',‘'counselling service’',‘'counseling service’',‘'patient famil’',‘'refer patien’',‘'referred patien’']	‘'Patient Family Counseling Referra’']	‘'we have made a referral to patient and family counseling ’',‘'a referral to patient and family counseling has been made ’',‘'i have made a referral to patient and family counseling ’']
12	1009	‘'menarch’',‘'ag’',‘'menarche ag’',‘'menopaus’',‘'menopause ag’',‘'history menarch’',‘'gynecologica’',‘'gynecological histor’',‘'histor’',‘'menstrua’']	‘'menarche ag’',‘'age menarch’',‘'gynecological histor’',‘'gynecologic histor’',‘'12 menopaus’',‘'14 menopaus’',‘'menarche 1’',‘'gynecologica’',‘'13 menopaus’',‘'15 menopaus’']	‘'Menstrual Histor’']	‘'gynecological history her menarche was at age 12 ’',‘'gynecological history menarche at age of 12 , menopause age 52 ’',‘'gynecological history menarche age 12 , menopause age of 50 ’']
13	669	‘'lung’',‘'clea’',‘'lungs clea’',‘'auscultatio’',‘'clear auscultatio’',‘'auscultation lung’',‘'bilaterall’',‘'clear lung’',‘'bilaterally lung’',‘'entr’']	‘'auscultation lung’',‘'auscultation lun’',‘'lungs clea’',‘'clear lung’',‘'lung’',‘'lun’',‘'unremarkable lung’',‘'lungs norma’',‘'normal lung’',‘'lungs soun’']	‘'Clear Lungs Auscultatio’']	‘'lungs were clear to auscultation ’',‘'his lungs are clear to auscultation ’',‘'her lungs are clear to auscultation ’']
14	495	‘'crania’',‘'cranial nerv’',‘'nerv’',‘'nerve’',‘'cranial nerve’',‘'norma’',‘'examinatio’',‘'intac’',‘'i’',‘'examination norma’']	‘'cranial nerv’',‘'neurological examinatio’',‘'neurologic examinatio’',‘'cranial nerve’',‘'neurological exa’',‘'nerve abnormalitie’',‘'normal examinatio’',‘'examination norma’',‘'crania’',‘'focal crania’']	‘'Normal Cranial Nerve Examinatio’']	‘'neurologic the cranial nerve exam for cranial nerves ii through xii was normal ’',‘'he has a normal cranial nerve examination ’',‘'normal cranial nerve examination ’']
15	375	‘'myelom’',‘'marro’',‘'bone marro’',‘'multiple myelom’',‘'marrow biops’',‘'bon’',‘'multipl’',‘'biops’',‘'diagnosis multipl’',‘'diagnosi’']	‘'marrow biops’',‘'biopsy bon’',‘'bone marro’',‘'biops’',‘'biopsy performe’',‘'marro’',‘'biopsy pendin’',‘'marrow aspirat’',‘'myelom’',‘'multiple myelom’']	‘'Bone Marrow Biopsy for Myelom’']	‘'bone marrow biopsy ’',‘'bone marrow biopsy ’',‘'she has not had a bone marrow biopsy ’']
16	308	‘'alcoho’',‘'smokin’',‘'drink’',‘'da’',‘'qui’',‘'cigarette’',‘'cigarettes da’',‘'pac’',‘'mont’',‘'year’']	‘'drink mont’',‘'drinks alcoho’',‘'alcoho’',‘'drink’',‘'drinkin’',‘'drinks da’',‘'alcoholis’',‘'liquo’',‘'alcohol rar’',‘'history alcoho’']	‘'alcohol consumption pattern’']	‘'alcohol one to two drinks per month ’',‘'alcohol three drinks a month ’',‘'alcohol two drinks per month ’']
17	204	‘'blood wor’',‘'wor’',‘'bloo’',‘'baselin’',‘'baseline bloo’',‘'work toda’',‘'toda’',‘'ce’',‘'obtai’',‘'marker’']	‘'baseline bloodwor’',‘'baseline bloo’',‘'blood wor’',‘'today bloo’',‘'routine bloo’',‘'today baselin’',‘'baseline la’',‘'baselin’',‘'work baselin’',‘'blood test’']	‘'Baseline blood work toda’']	‘'will get baseline blood work today ’',‘'we will get baseline blood work today ’',‘'we will get baseline blood work today ’']
18	201	‘'clear bilaterall’',‘'bilaterally lung’',‘'bilaterall’',‘'lung’',‘'lungs clea’',‘'clea’',‘'posteriorly anteriorl’',‘'anteriorly lung’',‘'bilaterally negativ’',‘'opacity lef’']	‘'bilaterally lung’',‘'lungs clea’',‘'anteriorly lung’',‘'lung’',‘'changes lung’',‘'pulmonar’',‘'clear bilaterall’',‘'bilaterall’',‘'bilatera’',‘'bilaterally periphera’']	‘'clear lungs bilaterall’']	‘'the lungs are clear bilaterally ’',‘'lungs are clear bilaterally ’',‘'lungs are clear bilaterally ’']
19	189	‘'plu’',‘'’',‘'’',‘'’',‘'’',‘'’',‘'’',‘'’',‘'’',‘'’']	‘'plu’',‘'’',‘'’',‘'’',‘'’',‘'’',‘'’',‘'’',‘'’',‘'’']	‘'plus document’']	‘'p ’',‘'p ’',‘'plus p ’']

Topic representations using Maximal Marginal Relevance, KeyBERT, and Open AI’s Chat GPT 3.5-Turbo, alongside representative sentences of each topic. One date was anonymized to preserve privacy.

[a] Topic “-1” represents outlier sentences.

Table ST10: Additional representations and example sentences from using our new interpretation technique with models trained to predict seeing a counsellor

Topic	Count	Representation	KeyBERT	OpenAI	Representative_Docs
-1[a]	17511	['chemotherapy', 'patient', 'history', 'medical', 'pain', 'radiation', 'oncology', 'today', 'treatment', 'scan']	['oncology consultation', 'medical oncology', 'oncology', 'clinic', 'chemotherapy teaching', 'chemotherapy', 'tumor', 'consultation', 'patient', 'cancer']	['Cancer treatment history']	['social history this patient attended clinic today with her husband .', 'consulting service radiation oncology consultation requested by diagnosis squamous cell carcinoma of the anal canal , clinical stage t2 n0 mx , pending ct scan tomorrow and a pet ct scan that has been requested within a couple of weeks .', 'consulting service medical oncology consultation requested by i had the pleasure of meeting with [FIRST LAST] in the medical oncology clinic today .']
0	6164	['mg', 'medications', 'tylenol', 'daily', 'dexamethasone', 'allergies', 'mg daily', 'hydromorphone', 'taking', 'pain']	['40 mg', '20 mg', '50 mg', '25 mg', '500 mg', '30 mg', 'prescribed', '300 mg', '100 mg', '150 mg']	['Medications and Allergies']	['medications methylphenidate 5 mg q .', 'candesartan 8 mg p .', 'coversyl 2 mg p .']
1	6161	['chemotherapy', 'treatment', 'patient', 'family', 'patient family', 'today', 'plan', 'cycles', 'given', 'start']	['start chemotherapy', 'concurrent chemotherapy', 'neoadjuvant chemotherapy', 'chemotherapy', 'chemotherapy radiation', 'medical oncology', 'treatment plan', 'weekly paclitaxel', 'oncology', 'treatment']	['chemotherapy treatment plan']	['dr . [LAST NAME] is planning 5 doses of palliative chemotherapy to start as soon as possible .', 'dr . [LAST NAME] discussed neoadjuvant chemotherapy , and specifically the brlatwac chemotherapy protocol with weekly paclitaxel for 4 cycles , followed by 4 cycles of ac chemotherapy .', 'after the surgery , we plan on adjuvant chemotherapy with either ac followed by paclitaxel and trastuzumab or tch chemotherapy regimen .']
2	6096	['pain', 'depression', 'history', 'right', 'past', 'abdominal', 'left', 'does', 'difficulty', 'abdominal pain']	['pain left', 'chest pain', 'present illness', 'depression', 'past medical', 'pain right', 'discomfort', 'history depression', 'pain', 'abdominal pain']	['Mild intermittent abdominal pain']	['she denies chest pain or pain elsewhere .', 'past medical history depression .', 'she has no pain anywhere .']
3	4840	['scan', 'ct', 'pet', 'ct scan', 'bone', 'pet scan', 'bone scan', 'staging', 'mri', 'showed']	['bone scan', 'scan bone', 'pet scan', 'ct scan', 'scan ct', 'mri scan', 'scan performed', 'scan chest', 'mri', 'scan']	['Scans for Bone Staging']	['bone scan and ct scan are pending .', 'we will get a pet scan .', 'with the pet scan , she will not need to have a bone scan .']
4	3620	['pack', 'smoking', 'years', 'alcohol', 'day', 'quit', 'pack year', 'history', 'cigarettes', 'ago']	['smoking history', 'year smoking', 'history smoking', 'quit smoking', 'tobacco', 'smoking', 'years quitting', 'smoke cigarettes', 'cigarettes', 'smoker']	['Smoking history and quitting']	['she quit smoking 6 years ago but was a 50 pack year smoker .', 'she has a 1 2 pack year smoking history , quit over 30 years ago .', 'social history she quit smoking 3 years ago and has a 45 pack year smoking history .']
5	2436	['cancer', 'breast', 'maternal', 'breast cancer', 'grandmother', 'old', 'year old', 'family history', 'paternal', 'family']	['cancer family', 'breast cancer', 'maternal grandmother', 'maternal grandfather', 'oncology history', 'family history', 'maternal aunt', 'cancer', 'grandmother', 'history maternal']	['Maternal Breast Cancer Family History']	['family history maternal grandmother had breast cancer .', 'family history her mother had breast cancer and grandmother also had breast cancer .', 'family history both her grandmother and her mother had breast cancer .']
6	2087	['works', 'social', 'social history', 'currently', 'lives', 'history', 'children', 'work', 'family', 'prince']	['nurse', 'mrs', 'teacher', 'worker', 'assistant', 'daughter', 'works office', 'lady', 'employed', 'administrative assistant']	['Multifaceted Work and Social History']	['she works in a nursery .', 'she works in a nursery with plants .', 'social history she works as a junior draftsperson .']
7[b]	2024	['', '', '', '', '', '', '', '', '', '']	['', '', '', '', '', '', '', '', '', '']	['n, n, d, r']	['n .', 'd .', 'r .']
8	1632	['pounds', 'lost', 'weight', 'weight loss', 'loss', 'months', 'appetite', 'pounds weight', '10', 'lost approximately']	['lost pounds', 'lost weight', 'weight loss', 'months weight', 'appetite lost', 'significant weight', 'pounds weight', 'weight months', 'weight', 'pounds']	['"Weight Loss Progress"']	['he has lost a few pounds in weight .', 'the 3 pounds of weight loss that she had lost she has regained .', 'weight 179 pounds .']

9	1178	['cranial', 'cranial nerve', 'normal', 'nerve', 'examination', 'oral cavity', 'cavity', 'oral', 'nerves', 'cranial nerves']	['nerve examination', 'examination cranial', 'neurological exam', 'cranial nerve', 'cranial nerves', 'neurologic exam', 'normal examination', 'examination normal', 'nerve abnormalities', 'exam normal']	['Normal Cranial Nerve Examination']	['normal cranial nerve exam .', 'normal cranial nerve exam .', 'neurologic the cranial nerve exam for cranial nerves ii through xii was normal .']
10	1160	['effects', 'fatigue', 'nausea', 'include', 'risk', 'alopecia', 'neutropenia', 'nausea vomiting', 'vomiting', 'limited']	['effects chemotherapy', 'febrile neutropenia', 'myelosuppression febrile', 'alopecia nausea', 'vomiting alopecia', 'acute effects', 'risk febrile', 'fatigue nausea', 'discussed effects', 'nausea']	['Side Effects of Chemotherapy']	['side effects of chemotherapy include but are not limited to nausea , vomiting , alopecia , mucositis , myelosuppression with rare episodes of febrile neutropenia , mucositis and fatigue .', 'side effects include , but are not limited to , nausea , vomiting , alopecia , mucositis , and myelosuppression with rare episodes of febrile neutropenia .', 'the side effects include but are not limited to fatigue , alopecia , nausea , vomiting , cardiomyopathy , peripheral neuropathy , and febrile neutropenia .']
11	848	['works', 'worked', 'work', 'currently', 'working', 'disability', 'occupation', 'driver', 'manager', 'worker']	['construction worker', 'worker', 'worker works', 'works carpenter', 'works chef', 'employed', 'laborer', 'occupation works', 'works manager', 'truck driver']	['Varied Occupations']	['he works as a boilermaker .', 'he works as a facilitator .', 'he works as a stockbroker .']
12	495	['menstrual', 'period', 'menstrual period', 'age', 'ago', 'menstrual cycle', 'gynecological', 'gynecological history', 'menopausal', 'premenopausal']	['menstruation', 'menstrual', 'menstrual period', 'menstruate', 'menstrual cycles', 'menstrual cycle', 'continues menstruate', 'period weeks', 'periods', 'period months']	['Menstrual history']	['her most recent menstrual period was this month .', 'her last normal menstrual period was october 6 .', 'menstrual history her last menstrual period was about a year ago .']
13	473	['memory', 'difficulty', 'able', 'term memory', 'short term', 'short', 'term', 'word', 'walk', 'word finding']	['memory impairment', 'memory difficulties', 'term memory', 'memory', 'memory loss', 'memory issues', 'recall', 'cognitive impairment', 'dementia', 'long term']	['Poor short-term memory difficulty']	['she has a very poor short term memory .', 'his short term memory is very poor .', 'she is able to name her family members but seems to have some difficulty with short term memory .']
14	345	['squamous', 'squamous cell', 'carcinoma', 'cell', 'cell carcinoma', 'diagnosis', 'differentiated', 'tongue', 'invasive', 'right']	['squamous carcinoma', 'cell carcinoma', 'carcinoma', 'carcinoma right', 'carcinoma left', 'cancer', 'carcinoma oncology', 'carcinoma situ', 'carcinoma p16', 'carcinoma consulting']	['Squamous Cell Carcinoma Diagnosed']	['squamous cell carcinoma of the base of tongue .', 'this has revealed a squamous cell carcinoma .', 'diagnosis t3 squamous cell carcinoma .']
15	199	['port', 'cath', 'port cath', 'require port', 'insertion', 'require', 'placement', 'cath insertion', 'inserted', 'cath inserted']	['port cath', 'port', 'cath inserted', 'need port', 'cath placement', 'arrange port', 'requires port', 'today port', 'cath insertion', 'cath']	['Port Cath Insertion']	['we will request a port a cath .', 'she has a port a cath in .', 'she also had a port a cath put in .']
16	182	['glioblastoma', 'glioblastoma multiforme', 'multiforme', 'grade', 'diagnosis', 'temporal', 'lobe', 'frontal', 'left', 'resection']	['glioblastoma multiforme', 'glioblastoma', 'grade glioblastoma', 'glioma', 'brain tumor', 'brain tumour', 'grade glioma', 'astrocytoma', 'grade astrocytoma', 'tumor']	['Glioblastoma Frontal Lobe']	['provisional diagnosis glioblastoma multiforme of the left frontal lobe .', 'diagnosis left temporal glioblastoma multiforme , who grade 4 .', 'oncology history and consultation diagnosis glioblastoma multiforme of the left frontal lobe .']
17	174	['depression depression', 'depression', 'trazodone', 'depression significant', 'significant alcohol', 'alcohol intake', 'intake', 'significant', 'alcohol', 'use depression']	['depression', 'depression depression', 'use depression', 'depression significant', 'depression sleep', 'alcohol', 'sleep', 'cause', 'unknown', 'alcohol use']	['Depression and alcohol use']	['depression .', 'depression .', 'depression .']
18	159	['respiratory', 'auscultation', 'air entry', 'entry', 'air', 'reveals', 'exam reveals', 'sounds', 'clear', 'exam']	['respiratory examination', 'respiratory', 'lungs', 'air entry', 'lung', 'breathing', 'bronchial', 'right lung', 'lung fields', 'chest exam']	['Clear Breath Sounds']	['respiratory exam reveals equal bilateral air entry to bases .', 'respiratory exam reveals equal bilateral air entry to bases .', 'her respiratory exam reveals bronchovesicular breath sounds and good air entry to the bases bilaterally .']

19	151	['tylenol tylenol', 'tylenol', 'half tab', 'tab', 'imodium', 'needed tylenol', 'break', 'tylenol needed', 'doses', 'half']	['tylenol', 'tylenol needed', 'tylenol tylenol', 'needed tylenol', 'imodium', 'doses', 'half tab', 'tab', 'break', '']	['Tylenol dosage and breaks']	['tylenol .', 'tylenol no .', 'tylenol and tylenol no .']

Topic representations using Maximal Marginal Relevance, KeyBERT, and Open AI's Chat GPT 3.5-Turbo, alongside representative sentences of each topic. One date was anonymized to preserve privacy. Names of physicians and patients were redacted to maintain privacy.

[a] Topic "-1" represents outlier sentences.

[b] Topic 7 represents single-character sentences. These represent
