# Supplementary material for: Predicting which patients with cancer will see a psychiatrist or counsellor from their initial oncology consultation document using natural language processing
Source: Commun Med (Lond). 2024 Apr 8;4:69. doi: 10.1038/s43856-024-00495-x (PMC11001970; doi:10.1038/s43856-024-00495-x)
Supplement: Supplementary file 2 — Reporting summary [file 43856_2024_495_MOESM2_ESM.pdf]

nature portfolio

Corresponding author(s): John-Jose Nunez

Last updated by author(s): Feb 17, 2024

# Reporting Summary

Nature Portfolio wishes to improve the reproducibility of the work that we publish. This form provides structure for consistency and transparency in reporting. For further information on Nature Portfolio policies, see our Editorial Policies and the Editorial Policy Checklist.

## Statistics

For all statistical analyses, confirm that the following items are present in the figure legend, table legend, main text, or Methods section.

| n/a | Confirmed | |
|---|---|---|
| ☐ | ☒ | The exact sample size (*n*) for each experimental group/condition, given as a discrete number and unit of measurement |
| ☐ | ☒ | A statement on whether measurements were taken from distinct samples or whether the same sample was measured repeatedly |
| ☐ | ☒ | The statistical test(s) used AND whether they are one- or two-sided<br>*Only common tests should be described solely by name; describe more complex techniques in the Methods section.* |
| ☒ | ☐ | A description of all covariates tested |
| ☐ | ☒ | A description of any assumptions or corrections, such as tests of normality and adjustment for multiple comparisons |
| ☐ | ☒ | A full description of the statistical parameters including central tendency (e.g. means) or other basic estimates (e.g. regression coefficient) AND variation (e.g. standard deviation) or associated estimates of uncertainty (e.g. confidence intervals) |
| ☐ | ☒ | For null hypothesis testing, the test statistic (e.g. *F*, *t*, *r*) with confidence intervals, effect sizes, degrees of freedom and *P* value noted<br>*Give P values as exact values whenever suitable.* |
| ☒ | ☐ | For Bayesian analysis, information on the choice of priors and Markov chain Monte Carlo settings |
| ☒ | ☐ | For hierarchical and complex designs, identification of the appropriate level for tests and full reporting of outcomes |
| ☐ | ☒ | Estimates of effect sizes (e.g. Cohen's *d*, Pearson's *r*), indicating how they were calculated |

*Our web collection on statistics for biologists contains articles on many of the points above.*

## Software and code

Policy information about availability of computer code

| | |
|---|---|
| Data collection | Our work was primarily that of data analysis; please see below regarding the software used, as some of it could be described as pertaining data collection. |
| Data analysis | The software we used for data analysis will be available upon publication at: https://github.com/jjnunez11/scar_nlp_psych<br>We used custom code written in Python 3., in addition to publicly available Python packages. The specific packages and their versions, as well as a Conda environment file, are available at the repository above. |

For manuscripts utilizing custom algorithms or software that are central to the research but not yet described in published literature, software must be made available to editors and reviewers. We strongly encourage code deposition in a community repository (e.g. GitHub). See the Nature Portfolio guidelines for submitting code & software for further information.

## Data

Policy information about availability of data

All manuscripts must include a data availability statement. This statement should provide the following information, where applicable:

- Accession codes, unique identifiers, or web links for publicly available datasets
- A description of any restrictions on data availability
- For clinical datasets or third party data, please ensure that the statement adheres to our policy

We are unable to share the initial oncology consultation documents used in this work due to their number and our inability to anonymize the confidential

information within them. The trained BoW models will be available upon publication on a public GitHub repository. Due to the possibility of neural models storing extractable private data, we are unable to share our trained neural models publicly, but may be able to share them with interested parties pending medical ethics and institutional approval, and will be interested in exploring federated learning approaches. Researchers interested in using this raw data can contact BC Cancer to make a request; more information is available at http://www.bccancer.bc.ca/health-professionals/professional-resources/bc-cancer-registry/request-registry-data.

## Research involving human participants, their data, or biological material

Policy information about studies with human participants or human data. See also policy information about sex, gender (identity/presentation), and sexual orientation and race, ethnicity and racism.

**Reporting on sex and gender**

We report the sex composition of our patient sample with respect to their sex as this was the data that was provided to us. We did not conduct sex-based analyses of our predictive models as we find it out of the scope of our current work , as sex-based differences in related predictive-performance have not been established. We believe this could be something interesting to investigate in the future, and wrote this in our discussion.

**Reporting on race, ethnicity, or other socially relevant groupings**

We do not report upon race or ethnicity in this work.

**Population characteristics**

We selected our study cohort from the 59,800 patients at BC Cancer starting cancer care between April 1, 2011 and December 30, 2016. Patients were seen for malignant disease or for non-malignant or precancerous disease requiring specialist cancer care. BC Cancer provides most cancer care in British Columbia, and is affiliated with all radiation oncologists and over 85% of medical oncologists in the province. BC Cancer provides care at 6 geographically diverse settings, and oversees systemic therapy at the majority of the smaller Community Oncology Network locations. We excluded participants with over 1 cancer diagnosis and required patients to have at least one valid medical or radiation oncologist consultation document within 180 days of diagnosis. After using this criteria, this left 47,625 patients, of which 25,428 were women (53.4%) and 22,197 were men (46.6%), with a mean age (SD) of 64.9 (13.7) years. For our prediction targets, 662 (1.4%) of patients saw a psychiatrist, while 10,034 (21.1%) saw a counsellor, within 12 months of the initial document being generated.

**Recruitment**

We did not directly recruit patients, but instead utilized clinical data as described above.

**Ethics oversight**

The University of British Columbia BC Cancer Research Ethics Board provided approval for this prognostic study, and exempted this work from requiring informed consent from participants as it was not feasible to obtain.

Note that full information on the approval of the study protocol must also be provided in the manuscript.

# Field-specific reporting

Please select the one below that is the best fit for your research. If you are not sure, read the appropriate sections before making your selection.

☒ Life sciences ☐ Behavioural & social sciences ☐ Ecological, evolutionary & environmental sciences

For a reference copy of the document with all sections, see nature.com/documents/nr-reporting-summary-flat.pdf

# Life sciences study design

All studies must disclose on these points even when the disclosure is negative.

**Sample size**

We did not perform a sample size calculation beforehand. The numbers needed to train predictive models can vary extensively, but a number above 1,000 is generally considered sufficient for many types of models, while models incorporating neural models can often benefit from training on at least 10,000 samples. We knew our dataset would be over 10,000 based on prior work using BC Cancer clinical data so proceeded without any sample calculations.

**Data exclusions**

We excluded participants with over 1 cancer diagnosis and required patients to have at least one valid medical or radiation oncologist consultation document within 180 days of diagnosis. This exclusion criteria was established ahead of time, and was utilized in prior, published work.

**Replication**

To ensure reproducibility and external validity of our results, we evaluated our trained models on an internal holdout set. We first randomly separated our data into training (70%), development (10%) and testing (20%) sets. We then tuned and developed our models using only the training and development sets. To generate the final results, we trained models on the training data multiple times, stopping after performance did not increase on the development set. We then evaluated this model on the test set. We conducted this 10 times for each model to estimate variance.

**Randomization**

To generate the random allotment for our train, development, and test sets, we utilized the last digit of the participant study IDs, which were in turn loosely based on BC Cancer IDs. We used this method to aid reproducibility and ensure the three sets were never contaminated.

**Blinding**

Blinding in the traditional sense is not applicable to this study. However, I would say that our use of a holdout set is quite similar to blinding. During the development of the code and models, we were "blind" to the holdout set that would be used for the final evaluation, to ensure that we could not over-fit our models to the test data. Only once all tuning and development was complete did we evaluate our models on the test set, ensuring we could not bias our development to this testset.

# Reporting for specific materials, systems and methods

We require information from authors about some types of materials, experimental systems and methods used in many studies. Here, indicate whether each material, system or method listed is relevant to your study. If you are not sure if a list item applies to your research, read the appropriate section before selecting a response.

## Materials & experimental systems

| n/a | Involved in the study |
|---|---|
| ☒ | ☐ Antibodies |
| ☒ | ☐ Eukaryotic cell lines |
| ☒ | ☐ Palaeontology and archaeology |
| ☒ | ☐ Animals and other organisms |
| ☐ | ☒ Clinical data |
| ☒ | ☐ Dual use research of concern |
| ☒ | ☐ Plants |

## Methods

| n/a | Involved in the study |
|---|---|
| ☒ | ☐ ChIP-seq |
| ☒ | ☐ Flow cytometry |
| ☒ | ☐ MRI-based neuroimaging |

## Clinical data

Policy information about clinical studies

All manuscripts should comply with the ICMJE guidelines for publication of clinical research and a completed CONSORT checklist must be included with all submissions.

| | |
|---|---|
| Clinical trial registration | This study did not use data from a clinical trial |
| Study protocol | This study did not use data from a clinical trial so a clinical trial protocol is not applicable. |
| Data collection | This study did not utilize recruitment, but instead used already existing clinical data at BC Cancer. Data was collected from all BC Cancer patients starting care between April 1, 2011 and December 30, 2016. As described above, the documents were generated after oncologists interacted with patients at any of the BC Cancer sites throughout British Columbia. |
| Outcomes | We predefined our primary outcomes as metrics related to the performance of our predictive models. We focused upon balanced accuracy and receiver-operating-curve area-under-curve as both of these metrics are commonly used, and account for class-imbalance. Our secondary outcome was investigating how models used words or phrases to come up with their prediction. |
